# Multilocus Species Trees Show the Recent Adaptive Radiation of the Mimetic *Heliconius* Butterflies

**DOI:** 10.1093/sysbio/syv007

**Published:** 2015-01-28

**Authors:** Krzysztof M. Kozak, Niklas Wahlberg, Andrew F. E. Neild, Kanchon K. Dasmahapatra, James Mallet, Chris D. Jiggins

**Affiliations:** ^1^Butterfly Genetics Group, Department of Zoology, University of Cambridge, CB2 3EJ Cambridge, UK; ^2^Laboratory of Genetics, Department of Biology, University of Turku, 20014 Turku, Finland; ^3^Department of Entomology, The Natural History Museum, London SW7 5BD, UK; ^4^Department of Biology, University of York, YO10 5DD Heslington, York, UK; and ^5^Department of Organismic and Evolutionary Biology, Harvard University, Cambridge, MA 02138, USA

**Keywords:** Amazonia, diversification rate, incongruence, Lepidoptera, Miocene, mimicry, multispecies coalescent

## Abstract

Müllerian mimicry among Neotropical Heliconiini butterflies is an excellent example of natural selection, associated with the diversification of a large continental-scale radiation. Some of the processes driving the evolution of mimicry rings are likely to generate incongruent phylogenetic signals across the assemblage, and thus pose a challenge for systematics. We use a data set of 22 mitochondrial and nuclear markers from 92% of species in the tribe, obtained by Sanger sequencing and *de novo* assembly of short read data, to re-examine the phylogeny of Heliconiini with both supermatrix and multispecies coalescent approaches, characterize the patterns of conflicting signal, and compare the performance of various methodological approaches to reflect the heterogeneity across the data. Despite the large extent of reticulate signal and strong conflict between markers, nearly identical topologies are consistently recovered by most of the analyses, although the supermatrix approach failed to reflect the underlying variation in the history of individual loci. However, the supermatrix represents a useful approximation where multiple rare species represented by short sequences can be incorporated easily. The first comprehensive, time-calibrated phylogeny of this group is used to test the hypotheses of a diversification rate increase driven by the dramatic environmental changes in the Neotropics over the past 23 myr, or changes caused by diversity-dependent effects on the rate of diversification. We find that the rate of diversification has increased on the branch leading to the presently most species-rich genus *Heliconius*, but the change occurred gradually and cannot be unequivocally attributed to a specific environmental driver. Our study provides comprehensive comparison of philosophically distinct species tree reconstruction methods and provides insights into the diversification of an important insect radiation in the most biodiverse region of the planet.

Visual mimicry provides an excellent system in which to study the origins of biodiversity, as the targets of selection are clearly identifiable and the role of natural selection in promoting adaptation and ultimately speciation can be directly observed (Bates 1863; Benson 1972; Mallet and Barton 1989; Sherratt 2008; Pfennig 2012). Studies of mimetic assemblages have been instrumental in explaining many biological phenomena, but testing hypotheses regarding the evolution of mimicry depends heavily on our knowledge of systematic relationships between the participating taxa, particularly where mimics are closely related (Ceccarelli and Crozier 2007; Wright 2011; Penney et al. 2012). Unfortunately, both strong selection on adaptive loci, which may facilitate adaptive introgression, and rapid radiation leading to incomplete lineage sorting (ILS), are likely to be common in many systems, but are especially prevalent in mimetic butterflies (Savage and Mullen 2009; Kubatko and Meng 2010; Kunte et al. 2011; Zhang et al. 2013b). These processes can significantly interfere with the estimation of phylogenies (Maddison and Knowles 2006; Linnen and Farrell 2008; Edwards 2009; Anderson et al. 2012).

A large body of recent work has been devoted to the issue of incongruence between the species tree (the true speciation history) and the gene trees evolving within (Anderson et al. 2012; Cutter 2013). The traditional approach of concatenating the total genetic evidence into a supermatrix to obtain a global estimate of the predominant phylogenetic signal and hidden support (Gatesy and Baker 2005), without consideration for the heterogeneity of individual partitions, has been to some extent superseded by multispecies coalescent (MSC) techniques (reviewed in: Edwards 2009; Knowles and Kubatko 2010; Anderson et al. 2012; Cutter 2013; Leaché et al. 2014). The majority of the new MSC algorithms are intended to model at least some of the sources of heterogeneity between different markers, most frequently focusing on the problem of ILS (e.g. Maddison and Knowles 2006; Heled and Drummond 2010), sometimes addressing hybridization (e.g., Gerard et al. 2011; Yu et al. 2011), and in at least one case modeling discordance without specifying its potential source (Larget et al. 2010). Although the supermatrix approach remains popular and serves as an effective approximation of the species diversification history in most cases, its ability to properly assess the degree of statistical support for phylogenies has been brought into question and contrasted with the potential of the MSC techniques to assess confidence more realistically (Edwards 2009; Knowles and Kubatko 2010). Heliconiini are an especially interesting subject for a systematic study, where the purported robustness of MSC tools to gene flow and other sources of incongruence can be tested with real data.

We estimate the phylogeny and investigate the link between the dynamics of speciation and macroevolutionary factors in the Neotropical butterfly tribe Heliconiini (Nymphalidae: Heliconiinae). Heliconiini are arguably the most thoroughly researched example of microevolution in the Neotropics, the most biologically diverse region of the world (Hoorn et al. 2010). They comprise the genus *Heliconius* and nine smaller genera, providing a spectacular example of a radiation where speciation is promoted by divergence in mimicry of aposematic wing patterns (Jiggins et al. 2001; Arias et al. 2008; Merrill et al. 2012). Heliconiini, and especially *Heliconius*, form Müllerian mimicry rings of distantly related toxic species, in which mimetic species share the cost of educating avian predators by evolving similar wing patterns. Thus, Heliconiini are an excellent system for the study of convergence from both genomic and organismal perspectives (e.g., Duenez-Guzman et al. 2009; Hines et al. 2011; Bybee et al. 2012; Heliconius Genome Consortium 2012; Martin et al. 2012; Pardo-Diaz et al. 2012; Jones et al. 2013; Nadeau et al. 2013; Supple et al. 2013; Arias et al. 2014). In addition to studies of wing pattern evolution, there has been a recent proliferation of comparative molecular and genomic studies of other traits including vision (Pohl et al. 2009), chemosensation (Briscoe et al. 2013) and cyanogenesis (Chauhan et al. 2013). A robust and stable molecular phylogeny is therefore especially desirable for this clade.

*Heliconius*, the most species-rich genus of Heliconiini, has the key features of an adaptive radiation (Schluter 2000; Glor 2010). All *Heliconius* are found in broadly similar Neotropical habitats (Brown 1981) and most of the species appear to originate on the eastern slope of the Central and North Andes (Rosser et al. 2012). *Heliconius* is far more diverse than the other nine genera of the tribe, and individual species show high disparity in their dietary adaptations (Benson et al. 1975; Merrill et al. 2013), and wing pattern diversity (Brown 1981; Jiggins 2008). Numerous field and laboratory experiments have demonstrated the adaptive value of wing patterns in protection from predators (Langham 2004). Importantly, divergence in wing pattern also leads to both pre- and post-mating reproductive isolation and hence contributes to speciation (Jiggins et al. 2001; Merrill et al. 2012). There is also evidence that ecological divergence in mating behavior, preference for microhabitat, and host plant use permit sympatric co-occurrence of closely related species (Gilbert 1991; Mallet and Gilbert 1995; Jiggins et al. 1997). There is also evidence for putative key innovations, most notably pollen feeding, which enables unusually long lifespans and underlies much of the unusual ecology of the group (Cardoso and Gilbert 2013). An expansion of olfactory receptor gene families (Briscoe et al. 2013) plays a likely role in specific host and food plant preferences (Benson et al. 1975; Boggs et al. 1981; Merrill et al. 2013). In summary, *Heliconius* have undergone a burst of diversification associated with adaptive changes, some of which directly cause reproductive isolation. Nonetheless, one characteristic of adaptive radiations that has not yet been demonstrated in *Heliconius* is a temporal burst of species diversification (Glor 2010; Moen and Morlon 2014).

An unusual feature of *Heliconius* is the prevalence and importance of gene flow and hybridization, leading to a controversy over the validity of traditional species concepts in the clade (Beltrán et al. 2002; Mallet et al. 2007). At least 26% of all species of Heliconiini occasionally produce interspecific hybrids in the wild (Mallet et al. 2007), and at least one species, *Heliconius heurippa*, has resulted from homoploid hybrid speciation from parental forms diverged millions of years ago (Mavárez et al. 2006; Jiggins 2008; Salazar et al. 2010). Genome sequencing has shown that mimetic diversity in the *Heliconius melpomene* and silvaniform (*Heliconius numata*) clades is also explained by adaptive introgression of genes regulating the aposematic wing patterns (Heliconius Genome Consortium 2012; Pardo-Diaz et al. 2012). In addition, neutral gene flow seems to be widespread, influencing as much as 40% of the genome between *H. melpomene* and *Heliconius cydno* (Martin et al. 2013).

Heliconiini systematics has a long history, starting with early morphological work (Emsley 1965; Brown 1981; Penz 1999), through allozymes (Turner et al. 1979) and a combination of morphological and ribosomal DNA-restriction data (Lee et al. 1992), to studies based on the sequences of mitochondrial and nuclear markers (Brower 1994b; Brower and Egan 1997; Beltrán et al. 2002, 2007; Cuthill and Charleston 2012; Massardo et al. 2014). The most comprehensive study to date by Beltrán et al. (2007) attempted to address some of the difficulties by incorporating many taxa (38 of 46 *Heliconius*, 59 of 77 Heliconiini), considering two individuals of most species, and sequencing two mitochondrial (*CoI/II* and *16S*) and four nuclear markers (*EF1α*, *Wg*, *Ap*, and *Dpp*). However, this data set is still potentially inadequate to address the challenges posed by Heliconiini systematics, as 3 of the loci (*16S*, *Ap*, and *Dpp*) were only sequenced for 12 representative species. Among the other three markers, 65% of the variable sites resided in the fast-evolving mitochondrial partition *CoI/II*, raising the possibility that the inferred relations are largely driven by the historical signal of the matriline. The relationships between *Heliconius*, *Eueides* and the other eight genera were not resolved with good support, as might be expected if most of the phylogenetically informative variation comes from a fast-evolving partition. Importantly, the individual data partitions were analyzed in concatenation. Despite these shortcomings, Beltrán and colleagues confirmed that the morphological and behavioral characteristics of the major clades do not correspond directly to their evolutionary history and that the traditionally recognized genera *Laparus* and *Neruda* are most likely nested within the crown genus *Heliconius*.

The importance of Müllerian mimicry as a driver of individual speciation events has been well-established (Mallet et al. 1998; Jiggins 2008) whereas the macroevolutionary processes governing the evolution of the group have been largely neglected in empirical studies (but see Etienne et al. 2012; Rosser et al. 2012). Precise understanding of the evolution of the mimicry rings, as well as associated processes such as hybridization, requires knowledge of the relative timing of the divergence events and motivated the most widely cited study of the molecular clock in Arthropoda (Brower 1994a). Mallet et al. (2007) created a chronogram from a partially unresolved tree, using a relaxed clock procedure and the *CoI/II* alignment. Importantly, the first dated phylogeny of Heliconiini is not calibrated to an absolute standard, making it impossible to make inferences about the relation of the diversification process and the contemporaneous geological and climatic events. A recent comparative study suggests that the majority of *Heliconius* lineages originated in the northeastern Andes and spread to other parts of the continent (Rosser et al. 2012). The cumulative results of over 200 systematic studies demonstrate that most South American tropical clades have experienced periods of significantly elevated net diversification rate in response to Andean orogenesis, alterations in the hydrology and sediment dynamics of the present-day Amazon Basin, as well as local and global climatic changes (Hoorn et al. 2010; Turchetto-Zolet et al. 2013). These processes can result in allopatry of incipient lineages, or in creation of new ecological niches and change to the species-level carrying capacity of the environment leading to ecological speciation (Brumfield and Edwards 2007; Hoorn et al. 2010). In particular, we can hypothesize that the diversification rate of Heliconiini increased during the periods of especially rapid Andean uplift around 23, 12 and 4.5 Ma (Gregory-Wodzicki 2000; Solomon et al. 2008; Hoorn et al. 2010; Rull 2011).

## Aims of the Study

Here we aim to resolve the species tree of Heliconiini radiation and generate a data set including nearly all of the currently valid species in the tribe, sampling intraspecific diversity across the range of many species, combining whole-genome sequencing with Sanger sequencing. We apply a wide range of phylogenetic methods to reconstruct the species tree, including supermatrix, coalescent and network approaches, which allow us to assess the strength of the underlying signal of speciation. The power of our combined approach is harnessed to elucidate the importance of marker heterogeneity for the final assessment of systematic relationships, while realistically estimating the support values for our chosen topology. We date the time of individual divergence events with confidence, permitting an analysis of diversification dynamics. We thus present a comprehensive study of macroevolutionary dynamics in a mimetic system that has been studied intensively at the microevolutionary level.

## Materials and Methods

### Taxon Sampling

We sampled 180 individuals, including 71 of the 77 species in all genera of Heliconiini and 11 outgroup species. The specimens came primarily from our collection at the University of Cambridge, with additional specimens shared by museums and private collectors (online Appendix S1, available on Dryad at http://dx.doi.org/10.5061/dryad.44b4j). We included five outgroup species from the sister tribe Acraeini (Wahlberg et al. 2009) and three from the related genus *Cethosia*. The diverse analyses used in this article require different sampling designs and the demands of all the techniques cannot be easily accommodated in a single data set. For example, the network analysis based on nucleotide distance produced much better supported and resolved trees when the 95% or more incomplete data from historical specimens were not used, whereas the various MSC techniques required the use of at least two individuals per species and had to be based only on taxa with intraspecific sampling (Fulton and Strobeck 2009; Heled and Drummond 2010). Thus, we distinguish four data sets. The complete data matrix includes all the data. The core data set excludes 14 individuals represented solely by short DNA fragments from historical specimens. The single-individual data set includes both modern and historical specimens, but with only the single best-sequenced individual per taxon. Finally, the *BEAST matrix contains only the 17 species of *Eueides* and *Heliconius* with extensive sampling of multiple representatives of each species.

### DNA Sequencing

We used 20 nuclear and 2 mitochondrial loci as markers (Supplementary Tables S1 and S2; online Appendix S1, available on Dryad at http://dx.doi.org/10.5061/dryad.44b4j). The selection includes the three classic molecular markers for Lepidoptera (*CoI/II*, *EF1α*, and *Wg*), two markers proposed by Beltrán et al. (2007) (*16S* and *Dpp*), eight new universal markers proposed by Wahlberg and Wheat (2008) (*ArgK*, *Cad*, *Cmdh*, *Ddc*, *Idh*, *Gapdh*, *Rps2*, and *Rps5*), and nine highly variable loci identified by Salazar et al. (2010) (*Aact*, *Cat*, *GlyRS*, *Hcl*, *Hsp40, Lm*, *Tada3*, *Trh*, and *Vas*). Additional *Heliconius*-specific primers were designed for *Cmdh*, *Gapdh*, and *Idh*. Details of the primers and PCR cycles are listed in Supplementary Table S1, available on Dryad at http://dx.doi.org/10.5061/dryad.44b4j. For most species, sequences of the three basic markers for multiple individuals were already published (Brower 1997; Beltrán et al. 2007; Wahlberg et al. 2009; Salazar et al. 2010), and data for 26 individuals came exclusively from GenBank (online Appendix S1, available on Dryad at http://dx.doi.org/10.5061/dryad.44b4j).

We generated Sanger sequences for 103 specimens (online Appendix S1, available on Dryad at http://dx.doi.org/10.5061/dryad.44b4j). DNA was isolated from approximately 50 μg of thorax tissue using the DNeasy Blood & Tissue kit (Qiagen, Manchester, UK). PCR was carried out in a total volume of 20 μl, containing 1× Qiagen Taq buffer (Manchester, UK), 2.5 mM MgCl_2_, 0.5 μM of each primer, 0.2 mM dNTPs, 1 unit bovine serum albumin, 0.5 unit Qiagen Taq-Polymerase and 1 μl of the DNA extract. The following program was executed on a G-Storm cycler (Somerton, UK): denaturation 5 min at 94°C; 35 cycles of 30 s at 94°C, 30 s at the annealing temperature and 90 s at 72°C; final extension for 10 min at 72°C. The results were visualized by electrophoresis in 1.5% agarose gel stained with 1% ethidium bromide. PCR products were cleaned using the ExoSAP-IT system (USB, Cleveland, OH): 60 min at 37°C; 20 min at 80°C. We used gel purification with the Nucleo Spin Extract II kit (Macherey-Nagel, Düren, Germany) as needed. Sanger sequencing reaction was carried out with the BigDye Terminator v. 3.1 (AB, Foster City, CA): 2 min at 94°C; 25 cycles of 10 s at 94°C, 5 s at 50°C and 4 min at 60°C. The products were sequenced with the ABI 3730xl DNA Analyzer at the Sequencing Facility, Department of Biochemistry, University of Cambridge. We manually inspected the traces in CodonCode v. 4.0.4 using PHRED for quality assessment (CodonCode Corporation 2012).

At the time of our Sanger sequencing effort, whole-genome data generated for other studies became available from 57 individuals in 27 common species (Heliconius Genome Consortium 2012; Briscoe et al. 2013; Supple et al. 2013; Dasmahapatra K.K., Mallet J., unpublished data) (online Appendix S1, available on Dryad at http://dx.doi.org/10.5061/dryad.44b4j). 100 bp reads were generated using the Illumina Genome Analyzer II platform with insert size of 300–400 bp. We performed *de novo* assembly of the short reads in the program Abyss v. 1.3 (Simpson et al. 2009). Based on previous studies (Salzberg et al. 2012; Briscoe et al. 2013) and preliminary results (Baxter S., personal communication), we chose k-mer length of 31, minimum number of pairs n=5 and minimum mean coverage c=2 as optimal settings. The 20 nuclear markers were mined from the assemblies by megaBLAST (Camacho et al. 2009) in Geneious v. 5.5.1 (Biomatters Ltd 2012) using reference sequences from the *H. melpomene* genome. The quality of the recovered sequences was assessed by alignment to previously generated amplicon sequences of the same loci from the same individuals.

Mitochondrial sequences could not be recovered by *de novo* methods, presumably because the large number of reads from the highly abundant mitochondrial DNA contained a large enough number of erroneous sequences to interfere with the assembly. We reconstructed whole mitochondrial genomes of 27 species by mapping to the *H. melpomene* reference (The Heliconius Genome Consortium 2012), using the default settings in the Genomics Workbench v. 5.5.1 (CLCBio 2012). These data were analyzed separately from the 21 locus mixed nuclear-mitochondrial alignment.

### DNA Sequencing: Historical Specimens

Short fragments of *CoI/II* and *EF1α* were sequenced from historical specimens up to 150 years of age, obtained from museum and private collections (online Appendix S1, available on Dryad at http://dx.doi.org/10.5061/dryad.44b4j) and processed in a vertebrate genetics laboratory to reduce the risk of contamination. Instruments and surfaces were cleaned with 5% bleach and irradiated with UV for 30 min prior to use. One to two legs were washed in water, immersed in liquid nitrogen in a test tube for 30 s and ground up, followed by an extraction into 20 μl of buffer using the QIAmp DNA Micro Kit (Qiagen, Manchester, UK). We treated every fifth extraction and every fifth PCR as a negative control with no tissue or DNA extract. PCRs were carried out in a 20-μl volume using 1 unit of Platinum HiFi Taq Polymerase (Invitrogen, London, UK) and 1× buffer, 2.5 mM MgCl_2_, 0.5 μM of each primer, 0.2 mM dNTPs, 1 unit bovine serum albumin, sterilized DNAse-free water and 1–5 μl of the DNA extract depending on concentration. To accommodate shearing of DNA with time, we designed and applied PCR primers spanning short fragments of 200–300 bp (online Appendix S2, available on Dryad at http://dx.doi.org/10.5061/dryad.44b4j). We carried out amplification, product clean up and sequencing as above, partially accounting for possible cross-contamination by blasting the results against GenBank.

### Alignment and Gene Tree Estimation

Alignments for each locus were generated in CodonCode v. 3 to account for inverted and complemented sequences, and improved using MUSCLE v. 3.8 (Edgar 2004). We visualized the alignments of the coding loci (all except the mitochondrial *16S* and the *tRNA-Leu* fragment in the *CoI/II* sequence) in Mesquite v. 2.75 (Maddison and Maddison 2011) and checked translated sequences for stop codons indicating errors. The whole mitochondrial sequences were aligned to the *Acraea issoria* and *H. melpomene* references (Heliconius Genome Consortium 2012) using the G-INS-i algorithm in MAFFT (Katoh 2002). The number of variable and parsimony informative sites was estimated for each locus in PAUP* v. 4 (Swofford 2002). Models of sequence evolution implemented in MrBayes (Ronquist and Huelsenbeck 2003) were selected in MrModelTest v. 2.3 (Posada and Crandall 1998; Nylander 2004) based on the AIC (Akaike 1974). Xia's test in DAMBE v. 4.0 (Xia and Xie 2001) demonstrated saturation in the third codon position of *CoI/II*, prompting us to treat the third codon position of the fast-evolving *CoI/II* locus as a separate partition when estimating the gene tree. The Leucine tRNA (*tRNA-Leu*) fragment occurring in the middle of *CoI/II* displays very low variability and thus was included in one partition with the slower evolving first and second codon positions. Individual gene trees were estimated in MrBayes v. 3.1, using four runs of one chain, 10 million Markov Chain Monte Carlo (MCMC) cycles sampled every 1000, and 2.5 million cycles discarded as burnin based on the average standard deviation of split frequencies becoming less than 0.01 and a plateau in the log-likelihood values (Ronquist and Huelsenbeck 2003). The mitochondrial genes were concatenated due to their shared history, but treated as separate partitions with distinct models. All gene trees were visualized with FigTree v. 1.4 (Rambaut 2009).

### Detection of Conflicting Signals

We investigated the cyto-nuclear discordance and other conflicts in the phylogenetic signal with several methods. To illustrate the global reticulate signal in the data, a NeighborNet network was built with the pairwise distances calculated under the F84 correction, the most complex model that could be fitted to the data in the program SplitsTree v. 4 (Kloepper and Huson 2008). We reduced the data set to a single best-sequenced individual per species to exclude the reticulations resulting from the expected recombination within species. Next, the topological disparity among individual loci was illustrated using Multi-Dimensional Scaling of pairwise Robinson–Foulds distance (Robinson and Foulds 1981) between the gene trees, as estimated by TreeSetViz v. 1.0 (Hillis et al. 2005) in Mesquite. Calculation of the RF required trimming the trees to the minimal set of 54 shared taxa from 27 species, using the R package APE (Paradis et al. 2004; R Development Core Team 2008). Finally, we investigated whether topologies and branch lengths of the individual loci are consistent enough to justify concatenation of the markers by means of a hierarchical likelihood-ratio test in Concaterpillar v. 1.5 (Leigh et al. 2008).

### Supermatrix Phylogenetics

We created a supermatrix of the 20 nuclear and 2 mitochondrial markers in Mesquite. To increase the efficiency of tree searches, optimal partitioning schemes for the complete and single-individual data sets were identified in PartitionFinder v. 1.1 (Lanfear et al. 2012), using the Bayesian Information Criterion (Schwarz 1978) and a greedy search, followed by selection of nucleotide substitution models. Lists of optimal partitions for ML and Bayesian analyses can be found in online Appendix S2, available on Dryad at http://dx.doi.org/10.5061/dryad.44b4j. The maximum-likelihood (ML) phylogeny was searched for under the GTRGAMMA model in RAxML v. 8.1 with 350 bootstrap replicates under the GTRCAT approximation (Stamatakis 2006), where the number of repetitions was determined by the bootstopping criterion (Stamatakis 2014). To explicitly test the likelihood of various hypotheses for Heliconiini phylogeny, several alternative topologies were created in Mesquite, representing previously identified groupings, as well as possible placements of the enigmatic genera *Cethosia*, *Laparus* and *Neruda* (Supplementary Table S4, available on Dryad at http://dx.doi.org/10.5061/dryad.44b4j) (Brower 1994; Brower and Egan 1997; Penz 1999; Penz and Peggie 2003; Beltrán et al. 2007; Mallet et al. 2007). We then re-estimated the ML tree using each topology as a constraint. The likelihood scores of the original and alternative trees were compared using the Shimodaira–Hasegawa (SH) test (Shimodaira and Hasegawa 1989) and the expected likelihood weights (ELW) based on 1000 bootstrap replicates (Strimmer and Rambaut 2002). We note that due to computational constraints, the assumptions of the SH test were violated by comparing only a subset of all the possible topologies (Shimodaira and Hasegawa 1989). A separate phylogeny was generated for the unpartitioned whole mitochondrial alignment in RAxML under the GTRGAMMA model with 1000 bootstrap replicates.

### Dating the Radiation

A Bayesian chronogram was estimated using the program BEAST v. 1.8 (Drummond et al. 2012). To avoid incorrect estimates of the substitution rate parameters resulting from the inclusion of multiple samples per species, this analysis was based on a pruned alignment with one individual per species. The only exception is the inclusion of three races of *H. melpomene* and two races of *Heliconius erato*, where deep geographical divergences are found (Quek et al. 2010). The Vagrantini sequences were not used, as BEAST can estimate the placement of the root without an outgroup (Drummond et al. 2012). Thus the analysis included 77 taxa and the optimal partitioning scheme was re-estimated appropriately (Table 1). We linked the topology, but modeled an uncorrelated lognormal clock and the substitution rate separately for each partition (Drummond et al. 2012). Substitution rates were drawn from the overdispersed gamma distribution prior with shape parameter k=0.001, scale parameter theta = 1 and starting value 0.001 for nuclear genes, and k=0.01 for the faster-evolving mitochondrial loci (van Velzen et al. 2013). We used a Birth–Death tree prior and empirical base frequencies to limit the computation time for the heavily parameterized model.

**Table 1.** Mean split ages in Ma at different levels of divergence within Heliconiini, as estimated by previous studies and by two Bayesian relaxed clock methods in the present work

	*Heliconius* versus *Eueides*	*Heliconius* versus *Agraulis*	*Heliconius* versus *Philaethria*	*melpomene* versus *erato*	*erato* versus *hecalesia*
Mallet et al. (2007)	11.0	13.0	14.5	9.5	4.0
Pohl et al. (2009)	n/a	∼32 (∼26–38)	n/a	∼17 (∼12–22)	n/a
Wahlberg et al. (2009)	18.5 (12.2–23.9)	26.5 (21.0–31.6)	30.0 (24.6–36.2)	n/a	n/a
Cuthill and Charleston (2012)	∼13 (∼10.5–16.5)	n/a	n/a	∼10.5(∼8.0–13.5)	∼4.5 (∼2.7–6.3)
BEAST	18.4 (16.5–20.6)	23.8(21.7–26.6)	26.2 (24.8–29.0)	11.8 (10.5–13.4)	4.4 (3.7–5.1)
*BEAST	17.5 (11.6–23.5)	n/a	n/a	10.5 (6.7–14.9)	n/a

*Note:* 95% credible intervals and highest posterior densities reported if known.

As no fossils of Heliconiini or closely related tribes are known, we used secondary calibration points from the dated phylogeny of Nymphalidae (Wahlberg et al. 2009; Mullen et al. 2011; van Velzen et al. 2013). Prompted by the findings of Sauquet et al. (2012), who demonstrate the potential for biases when secondary dating information is used, we compared having a single calibration point at the root with using all of the eight known split times. We also considered the impact of modeling each prior divergence time as normally distributed with the mean found by Wahlberg et al. (2009) and the standard deviation matching the 95% credible intervals, or as uniformly distributed within the same bounds. For each of the four model combinations, four independent instances of the MCMC chain were run for 100 million generations each, sampling the posterior every 10,000 generations and discarding 10 million generations as burnin after convergence assessment in Tracer v. 1.6 (Rambaut et al. 2014). Based on the marginal likelihood estimated under the AICM criterion (Baele et al. 2012), we chose the scheme with multiple calibrations modeled as uniformly distributed (Supplementary Table S3, available on Dryad at http://dx.doi.org/10.5061/dryad.44b4j), although we found that the ages of most of the nodes do not differ by more than 10% between the four schemes. While we recognize that the models should ideally be chosen based on the Path Sampling or Stepping Stone procedures (Baele et al. 2012), in practice we found these to be too computationally demanding for our relatively large data set. The input .xml file generated in BEAUti v. 1.8 (Drummond et al. 2010) can be found in the online Appendix S3, available on Dryad at http://dx.doi.org/10.5061/dryad.44b4j. To ensure that the results are driven by the data and not the priors, we executed an empty prior run. The Maximum Clade Credibility tree with mean age of the nodes was generated using LogCombiner v. 1.8 and TreeAnalyser v. 1.8 (Drummond et al. 2012). Parallel to the SH and ELW tests, plausible alternatives to the disputed and poorly supported nodes in the Bayesian chronogram (Supplementary Table S5, available on Dryad at http://dx.doi.org/10.5061/dryad.44b4j) were tested by calculating the posterior model ratios, a simple and robust alternative to the computationally expensive Bayes factor calculations (Bergsten et al. 2013). A posterior sample of 1000 phylogenies was filtered in PAUP* using constraint trees designed in Mesquite and the model odds were calculated as the frequency of the alternative grouping divided by the frequency of the clade observed in the MCC chronogram.

### Multispecies Coalescent Phylogenetics

To account for the heterogeneous phylogenetic signal resulting from gene flow, hybridization and ILS, we applied a variety of MSC analyses that take as input both the raw alignment and individual gene trees. We first used the established method of Minimizing Deep Coalescences (MDC) (Maddison and Knowles 2006), taking advantage of a dynamic programming implementation in the package PhyloNet (Than et al. 2008). One hundred samples of 100 trees were drawn randomly without replacement from the distribution of Bayesian gene trees for the 21 loci, an MDC phylogeny was estimated for each sample and a 50% majority rule consensus was taken.

Bayesian concordance analysis (BCA) is an MSC method that attempts to reconcile the genealogies of individual loci based on posterior distributions, regardless of the sources of conflict (Larget et al. 2010). BCA generates concordance factors (CFs), which show what proportion of loci contain a particular clade, and estimates the primary phylogenetic hypothesis from the best-supported clades. CFs offer a powerful alternative to traditional measures of support and can be conveniently estimated in the program BUCKy (Ané et al. 2007; Larget et al. 2010). We executed two runs of 1 million MCMC cycles in BUCKy based on the 21 posterior distributions of gene trees from MrBayes.

Another approach to the MSC is to estimate the gene trees and the species tree simultaneously, explicitly modeling the sources of incongruence (Edwards et al. 2007). We applied this technique using *BEAST, a program harnessing the power of BEAST and simultaneously implementing a powerful MSC algorithm that estimates the species tree and the embedded gene trees, as well as the population sizes of the lineages (Heled and Drummond 2010). Effective calculation of the population size parameters requires a thorough multilocus sampling of each species in the analysis, which forced us to reduce the data set to species with a minimum of three individuals from at least two distinct populations (the *BEAST data set). The final alignment included 87 terminal taxa in 17 species of *Eueides* and *Heliconius*. We re-estimated the individual substitution models for each partition (Supplementary Table S2, available on Dryad at http://dx.doi.org/10.5061/dryad.44b4j), used a constant population size coalescent tree model, and implemented other priors as described above for BEAST. We carried out four independent runs of 500 million cycles each, sampling every 10,000 cycles, generated a maximum clade credibility species tree and visually summarized the 21 gene trees by plotting in DensiTree (Bouckaert 2010). The online Appendix S4, available on Dryad at http://dx.doi.org/10.5061/dryad.44b4j, contains the .xml file for this analysis.

### Species Delineation

New species of Heliconiini continue to be described based on morphological, genetic, or karyotypic evidence (Lamas et al. 2004; Constantino and Salazar 2010; Dasmahapatra et al. 2010; Moreira and Mielke 2010), yet the validity of the taxonomic status of some of these new lineages has been disputed (Mallet J., Brower A., personal communication). To explore the potential for phylogenetic determination of the number of species in the tribe Heliconiini, species were delimited with the novel Bayesian Poisson Tree Process (bPTP) algorithm (Zhang et al. 2013b). Using the ML tree with multiple individuals per species, two runs of 500,000 generations were executed with the first 10% discarded as burnin based on the convergence of log-likelihood values. The results were often poorly supported and included clusters and splits not compatible with the extensive knowledge of the biology of this group (Supplementary Fig. S7, available on Dryad at http://dx.doi.org/10.5061/dryad.44b4j), prompting us instead to use the accepted taxonomy of Lamas et al. (2004) in our study of diversification dynamics in this clade.

### Changes in the Diversification Rate

The formal analyses of diversification dynamics were based on the output of the Bayesian supermatrix analysis in BEAST. Initially we investigated the changes in the number of lineages by generating semi-log lineage through time (LTT) plots for Heliconiini and *Heliconius* in the R package paleotree (R Development Core Team 2008; Bapst 2012) based on a posterior sample of 1000 chronograms, plotting the 95% credible interval of the divergence time around the median curve. We also carried out preliminary analyses in the R package DDD (Etienne and Haegemann 2012), using an MCC tree from an analysis with a single root calibration. Comparisons of all possible models, including diversity-dependence and changes in rate regimes, suggested that the diversification rate of Heliconiini increased around 12 Ma, shortly before the appearance of *Heliconius*, which in turn started to diversify faster around 4.5 Ma.

To achieve a more nuanced understanding of the diversification dynamic in this clade, we chose the reversible-jump MCMC (rjMCMC) approach implemented in the R package BAMM (Rabosky 2014; Rabosky et al. 2014a), which models both the variation in rates between lineages and the change of rates in time. BAMM is distinct from previously published approaches, as it also treats the location of diversification regimes on the tree as a variable to model (Rabosky 2014). In effect, the algorithm does not give false confidence in single, point estimates of rate shift times. To account for the uncertainty surrounding the influence of dating priors and the divergence times, we carried out analyses with four chronograms: the MCC phylogeny, the two trees corresponding to the upper and lower boundary of the 95% credible interval, as well as the MCC tree from an analysis with a single, normally distributed calibration at the root. All the phylogenies were truncated to 1 Ma before the present, to account for the uncertainty in the estimates of recent speciation (protracted speciation) and extinction rates (pull of the present; Etienne and Haegemann 2012). Appropriate speciation and extinction prior values were chosen for each tree in the BAMMtools suite (Rabosky et al. 2014b). We experimented with three alternative values of the Poisson process rate prior suitable for relatively small trees (1.0, 2.0, 4.0) and discovered that the results are very similar, as evidenced by insignificant differences in Bayes factors.

Following the failure of automated species delimitation, incomplete sampling of the taxa was accounted for by coding in the sampling proportion of each genus and each major *Heliconius* clade according to the taxonomy of Lamas et al. (2004), with addition of new species of *Philaethria* (Constantino and Salazar 2010) and *Neruda* (Moreira and Mielke 2010), and excluding *Heliconius tristero* (Mérot et al. 2013) (species sampling fraction = 0.904). A test with the most extreme of the plausible taxonomies (not recognizing *H. heurippa*, *Eueides emsleyi*, the undescribed *Agraulis* or the three recently proposed *Philaethria* species; sampling fraction = 0.956) demonstrated that the results are robust to the assumptions on the proportion of missing taxa. We assumed that rate changes may occur in increments of 0.5 Myr. For each of the four trees, two independent runs of four Metropolis-Coupled MCMC chains were executed for 2,200,000 generations and the first 200,000 generations were discarded as burnin. Convergence was assessed with the R package coda (Plummer et al. 2006) by plotting the log likelihood of all chains and calculating the effective sample size for log likelihood and the number of rate shifts. BAMMtools were used to analyze the posterior set of 2000 models; compare the frequently observed rate regimes with Bayes factors; identify the branches where rate shifts occurred; calculate the mean speciation, extinction and diversification rates for major clades; and plot the change of these parameters in time.

## Results

### Sampling and DNA Sequencing

We successfully combined three approaches to sequencing, which resulted in the high taxonomic coverage and intraspecific sampling necessary for the MSC methods, and a sufficient sampling of loci for each individual (online Appendix S1, available on Dryad at http://dx.doi.org/10.5061/dryad.44b4j). Most of the data set consists of Sanger sequences from 108 individuals, 26 of which were already included in GenBank. We also obtained two classic lepidopteran markers *CoI/II* and *EF1α* from respectively 13 and 11 out of 14 historical specimens, thus adding eight species of Heliconiini that have not been sequenced previously.

We capitalized on the availability of Illumina data by generating *de novo* assembly contigs for an additional 57 individuals. The N50 of the Abyss assemblies ranged from 552 to 1921 bp (average 1206) and all the nuclear markers were successfully recovered by megaBLAST from every assembly. Whole mitochondrial sequences of the same individuals were recovered by read mapping, with about a 400-bp stretch of the hypervariable control region (Heliconius Genome Consortium 2012) incomplete in some sequences. We obtained a depth of coverage over 100× and high confidence in the base calls due to the high copy number of mtDNA in the tissue. Finally, we included the sequences extracted from the *H. melpomene* genome, as well as the previously published mitochondrial sequence of *A. issoria* (Heliconius Genome Consortium 2012).

**Figure 1.**
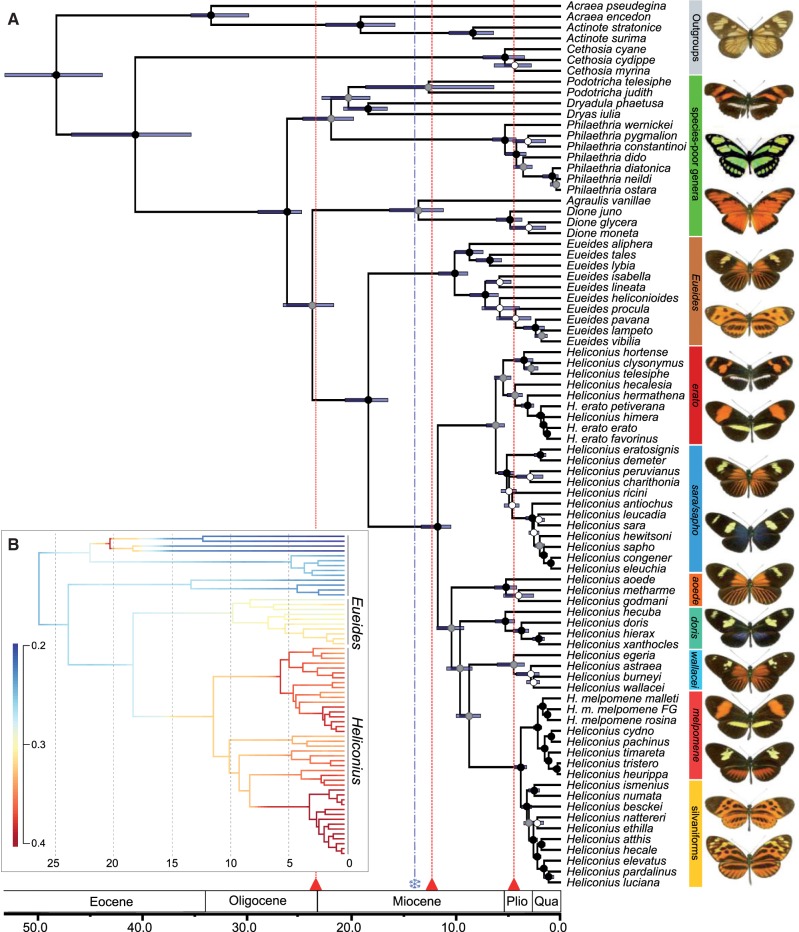
a) Bayesian phylogeny of 71 out of 77 butterflies in the tribe Heliconiini with outgroups, estimated using 20 nuclear and 2 mitochondrial markers with an uncorrelated molecular clock method (BEAST). The age of the root is calibrated based on the results of Wahlberg et al. (2009) and the bars signify the 95% credible intervals around the mean node ages. Scale axis in Ma. Deep splits are shown within the well-studied *H. erato* and *H. melpomene*. Heliconiini exhibit complex patterns of divergence and convergence in aposematic wing patterns, top to bottom: *Actinote latior* (outgroup), *P. telesiphe telesiphe*, *Philaethria dido chocoensis*, *Dione juno*, *Eueides tales michaeli*, *Eueides lampeto lampeto*, *Heliconius telesiphe telesiphe*, *H. erato favorinus*, *Heliconius demeter ucayalensis*, *H. sara sara*, *H. aoede cupidineus*, *H. doris* (blue morph), *H. burneyi jamesi*, *H. melpomene amaryllis*, *H. timareta contigua*, *H. numata lyrcaeus*, and *H. pardalinus dilatus*. b) The mean phylorate plot from the BAMM analysis. Net diversification rate is averaged across 2000 models fitted to the MCC chronogram. Colors from blue to red indicate the range of diversification rates from 0.2 to 0.4 new lineages per lineage per million years. Xaxis in millions of years ago. Photos © C. Jiggins, M. Joron and L. Constantino.

The sequence data for 20 nuclear and 2 mitochondrial genes encompass 70 out of 76 (92%) of the officially recognized species of Heliconiini, including 44 out of 46 species from the focal genus *Heliconius* (Lamas et al. 2004; Beltrán et al. 2007; Mallet et al. 2007; Constantino and Salazar 2010; Moreira and Mielke 2010; Mérot et al. 2013). Although the taxonomic validity of some species is contested, we found that the diversification analysis is robust to altering the number of missing species. Some recognized taxa diverged very recently, as shown by the BEAST chronogram (Fig. 1a) in case of the *Philaethria* *diatonica*/*Philaethria neildi*/*Philaethria ostara* complex and the *H. heurippa*/*H. tristero* pair, the latter of which was recently reclassified as a race of *Heliconius timareta* (Mérot et al. 2013). However, the exact relationship between genetic differentiation and taxonomic species identity in the highly variable, mimetic Heliconiini remains unclear (e.g., Mérot et al. 2013; Nadeau et al. 2013). Importantly, 36 species are represented by multiple individuals, usually from distant populations, allowing for more accurate estimation of sequence evolution rates, and detection of species paraphyly. The number of individuals represented by each marker ranges from 40% for *Hcl* to 98% for *CoI/II*, and only four specimens are represented exclusively by mitochondrial DNA (online Appendix S1, available on Dryad at http://dx.doi.org/10.5061/dryad.44b4j).

New DNA sequences were deposited in GenBank under accession numbers KP072800-KP074896 and KP113715-KP114069 (online Appendix S1, available on Dryad at http://dx.doi.org/10.5061/dryad.44b4j). Sequence alignments and phylogenetic trees were deposited in TreeBase (accession number 15531).

### Pervasive Conflict between the Loci

Our nuclear markers span 11 out of 21 chromosomes (Supplementary Table S2, available on Dryad at http://dx.doi.org/10.5061/dryad.44b4j; Heliconius Genome Consortium 2012) and have both autosomal and sex chromosome Z-linked inheritance. We examined the conflict between individual markers in the entire tribe and in the genus *Heliconius* alone, using both gene tree summary methods and approaches utilizing the raw sequence alignments. The ML analysis of the core matrix in Concaterpillar rejected concatenation of any of the loci due to significant differences in both topology and substitution rate of individual partitions, but the exact nature of the discordance is unclear. A Multi-Dimensional Scaling ordination of pairwise RF distances between the gene trees does not reveal clustering by chromosome and the separation between many nuclear loci appears much greater than between nuclear and mitochondrial trees (Supplementary Fig S1, available on Dryad at http://dx.doi.org/10.5061/dryad.44b4j). Consistent with this is the fact that the whole mitochondrial phylogeny of select taxa (Fig. 3) shows few differences from the tree based on the mixed marker supermatrix (Fig. 1a), highlighting that cytonuclear discordance is not the primary source of incongruence in the data set.

The coalescent approaches reveal the high extent of marker conflict in the *Heliconius* data. Gene tree topologies from the explicit Bayesian modeling of ILS in *BEAST are highly varied, with a particularly high degree of reticulation in the *H. melpomene*/*cydno* and the *Heliconius hecale* (silvaniform) clades, where extensive horizontal gene flow has been observed previously (Supplementary Fig. S2, available on Dryad at http://dx.doi.org/10.5061/dryad.44b4j; Brown 1981; Martin et al. 2013). Another Bayesian method, BUCKy, infers the species tree in the presence of marker incongruence without modeling specific reasons for the observed discordance, and calculates the Bayesian CFs that illustrate the proportion of partitions in the data set that support a particular grouping (Ané et al. 2007; Baum 2007; Larget et al. 2010). The concordance of the loci for *Heliconius* is strikingly low (Fig. 3), although the topology is consistent with the results of other analyses (Figs. 1a and 3; Supplementary Figs. S3–S7, available on Dryad at http://dx.doi.org/10.5061/dryad.44b4j).

Further strong evidence of widespread incongruence comes from the NeighborNet network characterized by a high delta score of 0.276, which shows that the structure of the data is not entirely tree-like (Supplementary Fig. S4, available on Dryad at http://dx.doi.org/10.5061/dryad.44b4j). This can be partially attributed to the effect of missing data, yet even a fit based on the 30 species with full or nearly complete sequences produced a delta score of 0.11, proving a substantial amount of non-bifurcating signal across the tribe (Holland et al. 2002). The most noticeable reticulations are found between nodes linking genera and the major clades of *Heliconius*, possibly due to pervasive gene flow during the diversification of the main extant lineages (Supplementary Fig. S4, available on Dryad at http://dx.doi.org/10.5061/dryad.44b4j).

### Topological Consistency across Optimality Criteria

Although no trees are identical, the results from our Bayesian, ML, and distance-based network analyses of the supermatrix are very similar (Fig. 1a, Supplementary Figs. S4 and S5, available on Dryad at http://dx.doi.org/10.5061/dryad.44b4j). Two basal nodes stand out as unstable. *Cethosia* is variably placed as a sister taxon to either Acraeini (MP, ML, NeighborNet) or Heliconiini (Bayesian), despite the reasonably extensive sampling of 11 loci for *Cethosia cyane*, while the position of *Podotricha* in relation to *Dryas* and *Dryadula* varies among all analyses. Most problematic are relations among the species within *Eueides*, where the position of 4 out of 10 taxa cannot be resolved with good support. The poor resolution for both *Eueides* and *Podotricha* can probably be attributed to insufficient site coverage, which produces high uncertainty due to patchily distributed missing data (Wiens and Morrill 2011; Roure et al. 2013). We re-estimated the relations of *Eueides* based solely on a core set of 11 genes with coverage for at least 7/10 species and recovered a much better supported tree (Supplementary Fig. S6, available on Dryad at http://dx.doi.org/10.5061/dryad.44b4j). We recommend studies focusing on *Eueides* use this specific phylogeny, but the exact relations of *Eueides* *procula*, *Eueides* *lineate*, and *Eueides* *heliconioides* remain unclear.

Although our Bayesian maximum clade credibility tree is similar to the topology estimated by Beltrán et al. (2007), significantly increasing the data set from 113 to 180 individuals and 5 to 21 loci allowed us to resolve many critical nodes. The major differences are in the relations of the genera other than *Heliconius*, which we infer to form a grade, with high support for *Eueides* as the sister genus of *Heliconius* (Fig. 1a). Importantly, we confirm that the enigmatic genera *Laparus* and *Neruda* are nested within *Heliconius*, as further supported by ELW and SH tests (Table 2), although *Neruda* is closer to the base of the genus than previously estimated (Fig. 1a). We find that the other so called “primitive” (Brown 1981) clade of *Heliconius* consists of two separate groups nested among other subgenera raising questions about the apparently unequal rate of morphological evolution in the genus.

**Table 2.** Speciation and extinction rate estimates for Heliconiini butterflies calculated in the BAMM analysis of macroevolutionary rates based on a posterior sample of 1000 BEAST chronograms

Clade	Speciation rate	Extinction rate
Heliconiini	0.267 (0.191–0.369)	0.130 (0.030–0.269)
*Heliconius*	0.318(0.215–0.450)	0.122 (0.012–0.281)
Non-*Heliconius*	0.236 (0.132–0.361)	0.135 (0.024–0.288)
*Eueides*	0.263 (0.125–0.396)	0.122 (0.012–0.275)
The eight species-poor genera	0.224 (0.111–0.356)	0.142 (0.024–0.316)

*Note:* Means and 90% highest posterior density intervals are reported for the focal clades and for all of Heliconiini excluding the most species-rich genus *Heliconius*.

The ML tree is similar to the Bayesian phylogeny, although it puts less confidence in the deeper nodes linking the genera and subgenera (Supplementary Fig. S5, available on Dryad at http://dx.doi.org/10.5061/dryad.44b4j), as is typically the case (Wiens and Morrill 2011). The nodes differing between the two trees are also the nodes that cannot be unequivocally confirmed by either the SH and the ELW test (Supplementary Table S4, available on Dryad at http://dx.doi.org/10.5061/dryad.44b4j). For instance, the highest posterior probability in the ELW test (0.302) is given to the tree that does not lump *Neruda* and the “primitive” *Heliconius*, consistent with the BEAST chronogram in Figure 1a. Thus, we suggest that the Bayesian tree should be preferred as a more accurate picture of the systematic relationships, although the ML phylogeny based on the complete data set is still useful to uncover multiple polyphyletic species. Notably, *Heliconius luciana* is nested within *Heliconius elevatus*, and *Heliconius wallacei* is polyphyletic with respect to *Heliconius astraea*. These results must be interpreted with caution, as the inference relies on poorly covered museum specimens and may be sensitive to long branch attraction (Wiens and Morrill 2011).

Our whole mitochondrial phylogeny is largely consistent with the results of the multilocus supermatrix analysis and well-supported for 46/57 nodes (Fig. 3), despite the relatively limited taxonomic coverage of only 29 Heliconiini for which short-read data were available. In contrast to the multilocus data set, mitochondrial genomes are not very useful for resolving relationships between major clades within *Heliconius*, as the positions of *Neruda*, *Heliconius* *xanthocles*, and *H. wallacei* clades are poorly supported. An important deviation from the predominantly nuclear multilocus phylogeny is the placement of *Heliconius pachinus* as a sister taxon of *H. timareta*, rather than *H. cydno*. This may reflect the overall instability resulting from a high extent of reticulation in the *melpomene*/*cydno*/*timareta* assemblage (Heliconius Genome Consortium 2012; Martin et al. 2013; Mérot et al. 2013). Furthermore, we find a surprising positioning of *Heliconius hermathena* within *H. erato* (Jiggins et al. 2008), which is also not supported by any of the analyses of the 21 locus matrix. The mitochondrial tree confirms previous observations of deep biogeographical splits in the widespread, highly diversified *H. erato* and its co-mimic *H. melpomene* (Brower 1994a). Within *H. melpomene* we find a well-supported distinction between races found to the west and east of the Andes, although our data place the individuals from French Guiana with the specimens from the Western clade, in contrast to a whole-genome phylogeny (Nadeau et al. 2013) (Fig. 1a). *Heliconius erato* shows the opposite pattern in both mitochondrial and nuclear data, whereby the Guianian races form a fully supported clade in the monophyletic group of taxa from East of the Andes.

The variety of MSC methods that we applied brings a new perspective to the phylogenetic signals in Heliconiini. We analyzed the small data set of the 17 best-sampled species with the Bayesian MSC algorithm implemented in *BEAST and recovered a tree which agrees with the supermatrix analyses (Supplementary Fig. S3, available on Dryad at http://dx.doi.org/10.5061/dryad.44b4j), except for the position of *Neruda* *aoede*, which was placed as a sister taxon to the *H. xanthocles*/*L. doris* clade with relatively low posterior probability. Furthermore, the mean ages of nodes are very close to those proposed in a supermatrix analysis (Table 1), with similar 95% credible intervals. Although the species tree is largely as predicted, we observe high levels of incongruence in the underlying distribution of gene trees (Supplementary Fig. S2, available on Dryad at http://dx.doi.org/10.5061/dryad.44b4j). Differences in the depth of coalescence are clear throughout the tree and reticulation is again especially apparent in the *H. melpomene/cydno* clade. The estimated population size values are also consistent with a previous comparison based on two nuclear loci, showing a higher population size of *H. erato* ($1.33\times 10^{6}$ individuals) when compared with *H. melpomene* ($1.0\times 10^{6}$) (Flanagan et al. 2004).

Similarly, we find the phylogeny derived by the gene tree summary approach BUCKy to be entirely consistent with the Bayesian analysis of concatenated sequence, although the recovered CFs are much lower than any other measure of support applied to our data. Importantly, most of the nodes connecting the major clades in the tree have CFs below 0.5, with the notable exception of the silvaniform/*melpomene* split (Fig. 3). The same nodes correspond to the reticulations in the NeighborNet analysis (Supplementary Fig. S4, available on Dryad at http://dx.doi.org/10.5061/dryad.44b4j), cases of low support in the MDC tree and its disagreement with the supermatrix analysis (Supplementary Fig. S7, available on Dryad at http://dx.doi.org/10.5061/dryad.44b4j), nodes that cannot be rejected in the ML and Bayesian tests of topologies (Supplementary Tables S4 and S5, available on Dryad at http://dx.doi.org/10.5061/dryad.44b4j), and the uncertain nodes in the whole mitochondrial tree (Fig. 3).

Another summary analysis by MDC, although taking only a few minutes with 100 bootstrap replicates of the complete data set, returns a very different topology from the other techniques, showing a number of unexpected and poorly supported groupings. The lumping of all non-*Heliconius* genera, and the monophyletic *Neruda*/*xanthocles*/*wallacei* clade stand out in contrast to other proposed trees (Supplementary Fig. S7, available on Dryad at http://dx.doi.org/10.5061/dryad.44b4j). Interestingly, many of the relations that are poorly supported in the supermatrix phylogenies are also not resolved in the consensus MDC tree, showing that MDC is highly conservative with regard to the placement of taxa unstable in individual gene trees.

### Tempo of Diversification

The phylogeny estimated under a relaxed clock model in BEAST shows diversification dynamics that differ from previous estimates, with the deeper splits between the genera of Heliconiini estimated as substantially older than previously inferred with mitochondrial data, but younger than estimated with a small sample of nuclear genes (Table 1) (Mallet et al. 2007; Pohl et al. 2009). The most species-rich genera *Heliconius* and *Eueides* separated 18.5 (95% highest posterior density: 16.5–20.4) Ma and both started to diversify, respectively, 11.8 (10.5–13.4) Ma and 10.2 (8.9–11.7) Ma. The six major clades of *Heliconius* (corresponding to *H. erato*, *Heliconius sara*, *H. xanthocles*, *H. wallacei*, *H. melpomene*/silvaniforms, and *Neruda*) all started to diversify around 5 Ma (Fig. 1b, Supplementary Fig. S8b, available on Dryad at http://dx.doi.org/10.5061/dryad.44b4j). The extinction rate has remained relatively constant throughout the history of the tribe, while the speciation rate (and thus net diversification) increased substantially in *Eueides* and *Heliconius* (Table 2). The LTT plot (Supplementary Fig. S8a, available on Dryad at http://dx.doi.org/10.5061/dryad.44b4j) and the plot of diversification rate for Heliconiini (Supplementary Fig. S9, available on Dryad at http://dx.doi.org/10.5061/dryad.44b4j) suggest rapid early emergence of *Podotricha*, *Dryadula* and *Dryas*, which later speciated at a very low rate (Table 2). This is followed by a period of stasis 18–12 Ma, roughly corresponding to the mid-Miocene (Hoorn et al. 2010), and a sudden but steady increase in the rate of diversification after 11 Ma. In the case of the 45 *Heliconius*, a shorter plateau is found between 9 and 6 Ma, and the number of extant lineages rises from 5 Ma onwards (Supplementary Fig. S8b, available on Dryad at http://dx.doi.org/10.5061/dryad.44b4j). As expected, the LTT plots drop off sharply in the last million years, reflecting protracted speciation (Etienne and Haegemann 2012).

ML modeling in DDD strongly supports the Birth–Death model with an increase in diversification rates 11 Ma as the best fit for Heliconiini (Akaike weight of 0.92; Supplementary Table S6, available on Dryad at http://dx.doi.org/10.5061/dryad.44b4j). However, we find that the confidence in the point estimate of shift time is largely a function of forcing the model over the data (Rabosky 2014). The Bayesian analysis with BAMM demonstrates that although there has likely been a large change in rate regimes across the phylogeny, it cannot be reduced to a single event at a strictly specified time, and this result is consistent for all four chronograms that we tested (values are reported for the MCC chronogram based on multiple uniform calibrations). Models with one or two shifts in diversification rate have the highest frequency in the posterior (0.430 and 0.281, respectively) and Bayes factors provide weak evidence for models with one shift (BF = 3.495) or two shifts (BF = 4.398) over models with no shift. Painting the mean rate per branch from 2000 individual models onto the phylogeny (Fig. 1b) and explicit testing of rate variation between clades (Supplementary Fig. S10, available on Dryad at http://dx.doi.org/10.5061/dryad.44b4j) show clearly that the rate changed on the branches leading to (i) the *Dryas* and *Podotricha* clade (BF >40); (ii) the *Heliconius* and *Eueides* clade (BF >10); and (iii) *Heliconius*. But even though there is evidence for rate increase on the *Heliconius* branch, no particular model with a specific shift time occurs at high frequency. On the contrary, the most frequent model (f=0.41) in the 95% credible set is the one with a constant rate.

## Discussion

### Stable Topology Despite Marker Incongruence

**Figure 2.**
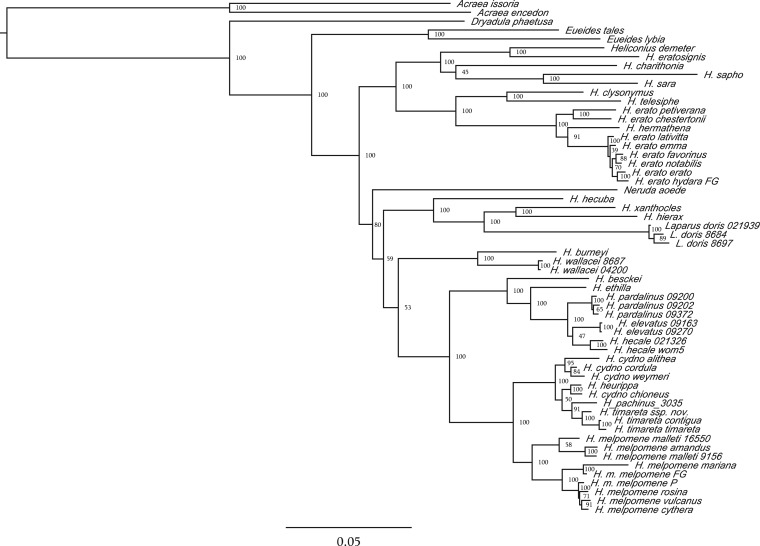
Whole mitochondrial ML (RAxML) phylogeny of the genus *Heliconius*. Bootstrap support values indicated. Scale bar in units of substitution per site per million years.

**Figure 3.**
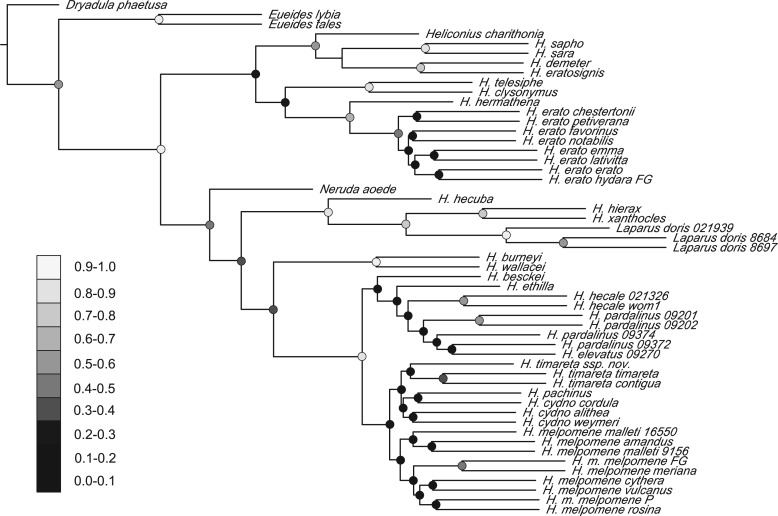
A phylogenetic hypothesis for *Heliconius* showing the extent of concordance between tree topologies of the 21 loci estimated by BCA in BUCKy. Dots indicate the CF values for the nodes, with darker shades of gray corresponding to lower support values.

We approached the problem of phylogeny reconstruction in a difficult mimetic assemblage through extensive intraspecific sampling of 22 markers from nearly all species in the clade, and compared the results between multiple philosophically distinct analytical approaches. As next generation sequencing technologies become widely accessible and the average number of loci used in systematic studies increases rapidly, MSC methods gain in importance as means of detecting and accounting for incongruence in multilocus data (e.g., Lee et al. 2012; Barker et al. 2013; Smith et al. 2013). However, their relative merits and utility at different levels remain contested (Song et al. 2012; Gatesy and Springer 2013; Reid et al. 2013). The systematic relations of the tribe Heliconiini, which diverged from its extant outgroup about 47 Ma, can be effectively resolved with both MSC and supermatrix approaches, yielding highly similar topologies across a range of different subsampling schemes that correspond to the requirements of individual algorithms (Figs. 1a and 3; Supplementary Figs. S3–S7, available on Dryad at http://dx.doi.org/10.5061/dryad.44b4j). Nonetheless, consistent with the recent radiation of the group, large effective population sizes and known hybridization between many species, we observed high heterogeneity among sampled fragments of the genome that differ markedly in both topology (Fig. 2, Supplementary Fig. S2, available on Dryad at http://dx.doi.org/10.5061/dryad.44b4j) and rates of evolution (Concaterpillar analysis). Such heterogeneity might have been expected to pose a significant challenge for the concatenation methods (Degnan and Rosenberg 2006; Edwards et al. 2007; Edwards 2009; Knowles and Kubatko 2010; Leaché and Rannala 2010). However, the only method producing an obviously different phylogeny is MDC (Supplementary Fig. S7, available on Dryad at http://dx.doi.org/10.5061/dryad.44b4j), which fails to resolve 34 out of 62 nodes in the tree with bootstrap support above 0.9, and is the only method to suggest monophyly of the non-*Heliconius* genera. MDC derives a species tree from point estimates of gene trees and can be expected to perform poorly with a relatively limited number of gene trees that are not always fully resolved, leading to a complete polytomy in some of the clades (Gatesy and Springer 2013). However, the MDC result is an indicator of instability, as well-resolved and consistent gene trees should produce a good quality MDC tree.

Recent studies have proposed that likelihood-based MSC techniques should be preferred to integration over individual gene trees, due to their potential to capture synergistic effects between partitions (Leaché and Rannala 2010; Reid et al. 2013), mirroring the phenomenon of hidden support in concatenation (Gatesy and Baker 2005). Our results support this observation and further show that high degrees of conflict between many partitions can be reconciled by both supermatrix and MSC approaches to extract the predominant signal of speciation. The BCA in BUCKy assigns insignificant CFs to most of the nodes separating major subgenera of *Heliconius*. Two of these nodes (*H. wallacei* and *N. aoede*; Fig. 3) are also only weakly supported by the *BEAST coalescent model, and correspond to the areas of high reticulation in the NeighborNet network, reflecting conflicting signals (Supplementary Fig. S4, available on Dryad at http://dx.doi.org/10.5061/dryad.44b4j). The same nodes are all assigned a posterior probability of one in the Bayesian supermatrix analysis (Fig. 1a), potentially leading to the erroneous conclusion that all the data point unequivocally to the inferred relations. The superiority of the MSC methods lies in the effective demonstration of incongruences, represented by lower support values or CFs assigned to the more difficult nodes (Belfiore et al. 2008).

### Optimal Sampling

Our study highlights an important practical consideration in choosing the optimal analytical approach, where the requirements of the selected algorithm have to be reconciled with a realistic sampling of taxa. Our final goal of testing hypotheses regarding the diversification dynamics of Heliconiini can be met only if the number of included taxa is maximized. Despite a substantial effort we only managed to secure single samples of the rare or geographically restricted species, and some of them are represented solely by historical specimens with limited potential to generate extensive multilocus data. Six other species remain missing, as they are known only from a small number of specimens (*Neruda metis*, *E. emsleyi*) or found at low density in infrequently visited areas (*Philaethria andrei*, *Philaethria browni*, *Philaethria romeroi*, and *Heliconius lalitae*). Specimens of four of these were available to us, but poor preservation of material by desiccation made it impossible to extract reasonable quality DNA. Considering that our focal group is intensively studied and exceptionally well represented in research, museum, and private collections due to its aesthetic appeal (Mallet et al. 2007), it would be considerably more challenging to obtain a complete sampling of many other groups. Another difficulty stems from the fact that the advanced coalescent techniques like BUCKy and *BEAST perform best with multiple samples per species, which should capture intraspecific diversity, and may require complete taxon coverage (Heled and Drummond 2010; Reid et al. 2013; Steel and Velasco 2014). Much of the uncertainty in the estimates can be attributed to missing data, which can negatively affect the estimation of both individual gene trees and the encompassing species tree (Wiens and Morrill 2011; Roure et al. 2013). When fitting a NeighborNet network, we found that although the percentage of data missing from the matrix does not explain all of the observed reticulation and the high delta score, it causes these parameters to increase, thus suggesting that the data completeness at each alignment position must be maximized to identify genuine incongruence. We observe that in many cases of biological interest it will be a formidable challenge to generate the ideal data set that (i) has little missing data, (ii) comprises a large, genome-wide sample of loci, (iii) includes all taxa, and (iv) captures intraspecific variability. In case of Heliconiini, the supermatrix approach based on a limited number of markers (22) helped us to maximize taxonomic inclusiveness without compromising our ability to reconstruct a phylogeny in the light of conflicting biological signals.

### Number of Species and Taxonomic Implications

Heliconiini have been at the center of the debate about species concepts and designation criteria, providing empirical evidence for the permeability of species barriers (Mallet et al. 2007; Nadeau et al. 2013; Kronforst et al. 2013; Martin et al. 2013). Species delimitation with the PTP based on relative branch lengths in the ML tree produced very surprising, although poorly supported results, strongly at odds with biological knowledge of Heliconiini. All the lineages in the *H. melpomene*/*H. cydno* group were lumped into a single Operational Taxonomic Unit (Supplementary Fig. S7, available on Dryad at http://dx.doi.org/10.5061/dryad.44b4j). This is despite extensive evidence for at least three species (*H. melpomene*, *H. cydno*, and *H. timareta*), if not the currently recognized five (also including *H. pachinus* and *H. heurippa*): premating behavioral isolation (Merrill et al. 2011), host plant usage and habitat preference (Benson et al. 1975; Brown 1981; Merrill et al. 2013), morphology (Penz 1999), and genome-wide genetic structure (Nadeau et al. 2013; Arias et al. 2014). The small number of nucleotide differences between the biological species is consistent with interspecific gene flow, which led to a reduction in differentiation across the genome. Although automated species delineation is certainly a useful criterion for initial assessment of poorly understood taxa or for rapid inventory of entire communities (Zhang et al. 2013b), we argue strongly that traditional taxonomies based on accumulation of extensive biological knowledge should take precedence in case of intensively researched taxa like Heliconiini. This is an important result, considering that the mitochondrial barcode region, although frequently used to delimit arthropod species at low cost, has also been shown to be insufficient to distinguish species in Ithomiini, a butterfly tribe co-mimicking Heliconiini (Elias et al. 2007).

Application of novel algorithms to Heliconiini data reveals a relatively stable topology and branch lengths, despite limited support (Figs. 1a and 3; Supplementary Figs. S2–S7, available on Dryad at http://dx.doi.org/10.5061/dryad.44b4j). We uphold the previous reclassification of the species in the genera *Neruda* (Hübner 1813) and *Laparus* (Linnaeus 1771) as Heliconius based on their nested position (Beltrán et al. 2007). This placement is at odds with morphological evidence from adult and larval characters, which puts *Neruda* and *Laparus* together with *Eueides* (Penz 1999), suggesting convergent evolution of homoplasious morphological characters. Nevertheless, the molecular evidence is decisive and we thus synonymize *Laparus* syn. nov. and *Neruda* syn. nov. with *Heliconius*. We also conclude that the traditional naming of some *Heliconius* as “primitive” (e.g., Brown 1981) is phylogenetically unjustifiable and misleading. Instead, we propose to call these groups “the *wallacei* clade” (including *H. astraea*, *Heliconius burneyi*, *Heliconius egeria*, and *H. wallacei*) and “the *doris* clade” (*Heliconius doris*, *Heliconius hecuba*, *Heliconius hierax*, and *H. xanthocles*).

The position of the enigmatic genus *Cethosia* remains unresolved, as it currently depends on the chosen method of analysis, and is unlikely to be established without a broad sampling of species using multiple markers. *Cethosia* has been variably considered to be either the only Old World representative of Heliconiini (Brown 1981; Penz and Peggie 2003; Beltrán et al. 2007), a genus of Acraeini (Penz 1999; Wahlberg et al. 2009), or possibly a distinct tribe (Müller and Beheregaray 2010). Establishing the systematic relations between Acraeini, *Cethosia*, and Heliconiini is important for the study of Heliconiini macroevolution, as it will shed light on the dispersal route of Heliconiini into the Neotropics.

### Divergence Time Estimates

Our phylogeny of Heliconiini brings novel insights into the diversification dynamics of the clade. Although most age estimates for *Heliconius* agree with other studies, the deeper nodes are older than previously suggested (Table 1). There is little agreement on the dates above the species level, and the studies to date either suffer from insufficient taxon sampling (Pohl et al. 2009; Wahlberg et al. 2009; Cuthill and Charleston 2012; Table 3), or use markers unlikely to be informative above a relatively low level of divergence (Mallet et al. 2007). For instance, the mean age of the split between *Heliconius* and *Agraulis* is estimated as 32 Ma in the study of Pohl et al. (2009), which includes only three species of Heliconiini; 26.5 Ma in Wahlberg et al. (2009), including one species per genus; or 23.8 Ma in the present study (Fig. 1a; Table 1). Conversely, Mallet et al. (2007) find the divergences between the genera of Heliconiini to be much younger than we propose, likely due to an effect of using a fast-evolving mitochondrial locus without partitioning (Brandley et al. 2011). Massardo et al. (2014) estimate broadly similar dates, but do not formally test alternative hypotheses of diversification and their conclusions are limited by incomplete sampling of the most diverse genera.

Our own ability to correctly falsify the hypotheses regarding the diversification of Heliconiini hinges on having a nearly complete phylogeny, yet none of our MSC analyses consider as many species of Heliconiini as the Bayesian supermatrix estimate. We are confident that the values proposed by the supermatrix method can be trusted, as both the topology and the branch lengths are consistent with the results inferred by *BEAST based on a smaller data set (Table 1; Supplementary Fig. S3, available on Dryad at http://dx.doi.org/10.5061/dryad.44b4j). We find that the ages of the deeper nodes and the length of terminal branches are not inflated by the supermatrix method in comparison to *BEAST, contrary to the predictions from simulations (Burbrink and Pyron 2011), and both methods infer similar mean age for the observed splits, for instance 11.8 Ma for the basal divergence of *Heliconius* into the *H. erato* and *H. melpomene* lineages, or around 3.5 Ma for *H. doris* and *H. xanthocles*. Hence, we offer a new perspective on the dating of the Heliconiini radiation with a nearly complete set of divergence time estimates based on a well-resolved and supported tree.

### *Adaptive but not Rapid Radiation of* Heliconius

Despite evolving many properties unique among the Lepidoptera (reviewed by Beltrán et al. 2007), *Heliconius* has not undergone a rapid adaptive radiation involving an explosive growth in the number of lineages, followed by a dramatic slowdown, as might be expected due to a sudden availability and filling of ecological niches (Pybus and Harvey 2000; Glor 2010; Moen and Morlon 2014). In fact, the rate of diversification has been increasing steadily for the last 11 myr (Supplementary Fig. S10, available on Dryad at http://dx.doi.org/10.5061/dryad.44b4j). This result is consistent with a recent critique of the traditional prediction that large radiations should display a pattern of the initially high net diversification rate decreasing as the ecological niches fill up (Day et al. 2013). Such an expectation is reasonable for island radiations that constitute a large proportion of study cases to-date, but it does not necessarily apply to continental radiations in the tropics, where the scale and complexity of the ecosystems are likely to generate a number of suitable niches greatly exceeding even the cladogenetic potential of large radiations (Derryberry et al. 2011; Day et al. 2013). To date, steady diversification of a widely distributed taxon has been demonstrated in the 129 African *Synodontis* catfish (Day et al. 2013) and the 293 Neotropical Furnariidae ovenbirds (Derryberry et al. 2011), but the generality of this pattern remains unknown and requires further verification in other well-sampled large groups like Heliconiini.

Both ML (DDD) and Bayesian (BAMM) approaches provide evidence for substantial increases in speciation without changes in extinction rate, although we cannot completely exclude the possibility that extinction has not been modeled accurately and may have affected our findings. The first surge in speciation occurred early on the branch leading to the presently depauperate clade of *Podotricha telesiphe*, *Podotricha* judith, and the monotypic genera *Dryas* and *Dryadula*, and stands out against the low overall background rate of diversification (Fig. 1b, Supplementary Fig. S10, available on Dryad at http://dx.doi.org/10.5061/dryad.44b4j). More substantial increases are observed in the *Heliconius* and *Eueides* clade (*Heliconius* mean net diversification: 0.196 new lineages per lineage per myr; non-*Heliconius* rate: 0.101 (Fig. 1b, Supplementary Fig. S11, available on Dryad at http://dx.doi.org/10.5061/dryad.44b4j). Bayesian modeling shows that the gains have been gradual and cannot be correlated with a single environmental factor, although the speciation of *Heliconius* and *Eueides* may have been stimulated by the second rapid stage of Andean orogeny approximately 12 Ma (Rull 2011), which strongly changed the elevation gradients in the Central and Eastern sectors (Gregory-Wodzicki 2000; Blandin and Purser 2013). This was contemporaneous with climatic changes that affected the distribution of the rainforest (Lewis et al. 2007; Jaramillo et al. 2010), and followed shortly by the entrenchment of a major barrier, the Amazon, in its modern course 10 Ma (Hall and Harvey 2002; Hoorn et al. 2010). Nonetheless, our results do not strongly support such a correlation and highlight the dangers (Rabosky 2014) of the commonly used approach of selecting a single rate shift configuration (reviewed in Stadler 2013). For example, analysis of the Heliconiini data with DDD provided false confidence in a single distinct shift to a much higher rate of diversification. The lack of strong response by Heliconiini to the environmental perturbations is less surprising if we consider that intense changes on the continental scale have occurred nearly continuously from the start of Miocene (and thus Heliconiini) until the present. This has created a dynamic arena for constant evolution of new species and colonization of novel ecological niches (Derryberry et al. 2011), across a variety of environmental regimes, ecosystems, and geological formations (Blandin and Purser 2013).

More puzzling is the gradual rise in the diversification rate of Heliconiini, clearly driven by the increased speciation rates of *Heliconius* (Fig. 1b, Supplementary Fig. S10, available on Dryad at http://dx.doi.org/10.5061/dryad.44b4j). One distinct possibility is that the extraordinary diversity of hundreds of mimetic patterns in the genus (see Fig. 1a for some examples) has facilitated speciation (Elias et al. 2008; Jiggins 2008; Merrill et al. 2012) and stabilized extinction (Vamosi 2005), leading to a positive feedback of higher number of species on the diversification rate. However, testing any ideas on the macroevolutionary impact of the mimetic phenotypes will be difficult. For instance, direct optimization of the evolution of color patterns on the phylogeny proved fruitless, as the signal of ancestral morphological states is lost behind the existing intraspecific diversity of widely varied races and the confounding effects of pattern elements introgressing between species (Beltrán 2004).

Some uncertainty surrounds the last few million years of evolution of Heliconiini. Early speculation regarding the drivers of speciation suggested diversification in allopatry, as the rainforest habitat occupied by most species in the group has undergone cycles of contraction and expansion in response to recent climatic variation (Turner 1965; Brown et al. 1974; Brown 1981; Sheppard et al. 1985; Brower 1994a). The hypothesis of vicariant cladogenesis has been subsequently criticized due to a lack of evidence for forest fragmentation in pollen core data (Colinvaux et al. 2000), lack of temporal concordance of divergence of distinct lineages (Dasmahapatra et al. 2010), and the likelihood of parapatric speciation (Mallet et al. 1998). The decrease in observed diversification over the last million years may be due to protracted speciation (Etienne and Haegemann 2012), that is, our limited ability to delineate species in the assemblages of highly variable taxa such as *Heliconius*.

In conclusion, we present a taxonomically comprehensive phylogeny of a large continental radiation characterized by extensive introgression and varying rate of diversification. Reticulate signals in phylogenetic data will increasingly have to be accounted for in study designs and could be common in other recent adaptive radiations, as recently reported for swordfish (Cui et al. 2013), mosquitoes (Crawford et al. 2014), or broomrape plants (Eaton and Ree 2013). Ignoring the possibility of reticulation no longer seems a viable assumption in phylogenetic analysis of recent adaptive radiations.

## Author Contributions

Conceived and designed the study: K.M.K., N.W., and C.D.J. Carried out the labwork, analyzed the data and drafted the manuscript: K.M.K. Provided samples and next-gen sequence data: C.D.J, J.M, K.K.D., N.W., and A.N. All authors have read and approved the final version of the manuscript.

## Supplementary Material

Data available from the Dryad Digital Repository: http://dx.doi.org/10.5061/dryad.44b4j.

## Funding

This work was supported by the Harvard Herchel Smith Trust, Emmanuel College and the Balfour Studentship from the Department of Zoology, Cambridge University [to K.M.K.]; the Leverhulme Trust Leadership Grant and the BBSRC grant H01439X/1 [to C.D.J.]; Kone Foundation [to N.W.]; the BBSRC [grant BB/G006903/1 to J.M. and K.K.D.].

## References

[B1] Akaike H. (1974). A new look at the statistical model identification. IEEE Trans. Automat. Contr..

[B2] Anderson C.N.K., Liu L., Pearl D., Edwards S.V. (2012). Tangled trees: the challenge of inferring species trees from coalescent and noncoalescent genes. Methods Mol. Biol..

[B3] Ané C., Larget B., Baum D.A., Smith S.D., Rokas A. (2007). Bayesian estimation of concordance among gene trees. Mol. Biol. Evol..

[B4] Arias C.F., Muñoz A.G., Jiggins C.D., Mavárez J., Bermingham E., Linares M. (2008). A hybrid zone provides evidence for incipient ecological speciation in *Heliconius* butterflies. Mol. Ecol..

[B5] Arias C.F., Salazar C., Rosales C., Kronforst M.R., Linares M., Bermingham E., McMillan W.O. (2014). Phylogeography of *Heliconius cydno* and its closest relatives: disentangling their origin and diversification. Mol. Ecol..

[B6] Baele G., Lemey P., Bedford T., Rambaut A., Suchard M.A., Alekseyenko A.V. (2012). Improving the accuracy of demographic and molecular clock model comparison while accommodating phylogenetic uncertainty. Mol Biol Evol..

[B7] Bapst D.W. (2012). paleotree: an R package for paleontological and phylogenetic analyses of evolution. Methods Ecol. Evol..

[B8] Barker F.K., Burns K.J., Klicka J., Lanyon S.M., Lovette I.J. (2013). Going to extremes: contrasting rates of diversification in a recent radiation of new world passerine birds. Syst. Biol..

[B9] Bates H.W. (1863). The naturalist on the river Amazons.

[B10] Baum D.A. (2007). Concordance trees, concordance factors, and the exploration of reticulate genealogy. Taxon.

[B11] Belfiore N.M., Liu L., Moritz C. (2008). Multilocus phylogenetics of a rapid radiation in the genus *Thomomys* (Rodentia: Geomyidae). Syst. Biol..

[B13] Beltrán M., Jiggins C.D., Brower A.V.Z., Bermingham E., Mallet J. (2007). Do pollen feeding, pupal-mating and larval gregariousness have a single origin in *Heliconius* butterflies? Inferences from multilocus DNA sequence data. Bio. J. Linn. Soc..

[B14] Beltrán M., Jiggins C.D., Bull V., Linares M., Mallet J., McMillan W.O., Bermingham E. (2002). Phylogenetic discordance at the species boundary: comparative gene genealogies among rapidly radiating *Heliconius* butterflies. Mol. Biol. Evol..

[B15] Benson W.W. (1972). Natural selection for Müllerian mimicry in *Heliconius erato* in Costa Rica. Science.

[B16] Benson W.W., Brown K.S., Gilbert L.E. (1975). Coevolution of plants and herbivores: passion flower butterflies. Evolution.

[B17] Bergsten J., Nilsson A.N., Ronquist F. (2013). Bayesian tests of topology hypotheses with an example from diving beetles. Syst. Biol..

[B18] http://www.geneious.com.

[B19] Blandin P., Purser B. (2013). Evolution and diversification of Neotropical butterflies: insights from the biogeography and phylogeny of the genus *Morpho* Fabricius, 1807 (Nymphalidae: Morphinae), with a review of the geodynamics of South America. Tropical Lepidoptera Research.

[B20] Boggs C.L., Smiley J.T., Gilbert L.E. (1981). Patterns of pollen exploitation by *Heliconius* butterflies. Oecologia.

[B21] Bouckaert R.R. (2010). DensiTree: making sense of sets of phylogenetic trees. Bioinformatics.

[B22] Brandley M.C., Wang Y., Guo X., Oca A.N.M., Fería-Ortíz M., Hikida T., Ota H. (2011). Accommodating heterogenous rates of evolution in molecular divergence dating methods: an example using intercontinental dispersal of *Plestiodon* (*Eumeces*) lizards. Syst. Biol..

[B23] Briscoe A.D., Muños A.M., Kozak K.M., Walters J.R., Yuan F., Jamie G.A., Martin S.H., Dasmahapatra K., Ferguson L.C., Mallet J., Jacquin-Joly, Emmanuelle Jiggins C.D. (2013). Female behaviour drives expression and evolution of gustatory receptors in Butterflies. PLoS Genet.

[B24] Brower A.V. (1994a). Rapid morphological radiation and convergence among races of the butterfly *Heliconius erato* inferred from patterns of mitochondrial DNA evolution. Proc. Natl. Acad. Sci. USA..

[B25] Brower A.V.Z. (1994b). Phylogeny of *Heliconius* butterflies inferred from mitochondrial DNA sequences (Lepidoptera: Nymphalidae). Mol. Phylogenet. Evol..

[B26] Brower A.V.Z. (1997). The evolution of ecologically important characters in *Heliconius* butterflies (Lepidoptera: Nymphalidae): a cladistic review. Zoo. J. Linn. Soc..

[B27] Brower A.V.Z., Egan M.G. (1997). Cladistic analysis of *Heliconius* butterflies and relatives (Nymphalidae: Heliconiiti): a revised phylogenetic position for Eueides based on sequences from mtDNA and a nuclear gene. Proc. R. Soc. B Biol. Sci..

[B28] Brown K., Sheppard P., Turner J. (1974). Quaternary refugia in tropical America: evidence from race formation in *Heliconius* butterflies. Proc. R. Soc. Lond. Ser. B Biol. Sci..

[B29] Brown K.S. (1981). The biology of *Heliconius* and related genera. Annu. Rev. Entomol..

[B30] Brumfield R.T., Edwards S.V. (2007). Evolution into and out of the Andes: a Bayesian analysis of historical diversification in *Thamnophilus* antshrikes. Evolution.

[B31] Burbrink F.T., Pyron R.A. (2011). The impact of gene-tree/species-tree discordance on diversification-rate estimation. Evolution.

[B32] Bybee S.M., Yuan F., Ramstetter M.D., Llorente-Bousquets J., Reed R.D., Osorio D., Briscoe A.D. (2012). UV photoreceptors and UV-yellow wing pigments in *Heliconius* butterflies allow a color signal to serve both mimicry and intraspecific communication. Am. Nat..

[B33] Camacho C., Coulouris G., Avagyan V., Ma N., Papadopoulos J., Bealer K., Madden T.L. (2009). BLAST+: architecture and applications. BMC Bioinformatics.

[B34] Cardoso M.Z., Gilbert L.E. (2013). Pollen feeding, resource allocation and the evolution of chemical defence in passion vine butterflies. J. Evol. Biol..

[B35] Ceccarelli F.S., Crozier R.H. (2007). Dynamics of the evolution of Batesian mimicry: molecular phylogenetic analysis of ant-mimicking *Myrmarachne* (Araneae: Salticidae) species and their ant models. J. Evol. Biol..

[B36] Chauhan R., Jones R., Wilkinson P., Pauchet Y., Ffrench-Constant R.H. (2013). Cytochrome P450-encoding genes from the *Heliconius* genome as candidates for cyanogenesis. Insect Mol. Biol..

[B37] http://www.clcbio.com/products/clc-genomics-workbench/.

[B38] http://www.codoncode.com/.

[B39] Colinvaux P.A., De Oliveira P.E., Bush M.B. (2000). Amazonian and neotropical plant communities on glacial time-scales: the failure of the aridity and refuge hypotheses. Quat. Sci. Rev..

[B40] Constantino L.M., Salazar J.A. (2010). A review of the Philaethria dido species complex (Lepidoptera: Nymphalidae: Heliconiinae) and description of three new sibling species from Colombia. Zootaxa.

[B41] Crawford J., Riehle M.M., Guelbeogo W.M., Gneme A., Sagnon N., Vernick K.D., Nielsen R., Lazzaro B.P. (2014). Reticulate speciation and adaptive introgression in the *Anopheles gambiae* species complex. bioRxiv.

[B42] Cui R., Schumer M., Kruesi K., Walter R., Andolfatto P., Rosenthal G.G. (2013). Phylogenomics reveals extensive reticulate evolution in *Xiphophorus* fishes. Evolution.

[B43] Cuthill J.H., Charleston M. (2012). Phylogenetic codivergence supports coevolution of mimetic *Heliconius* butterflies. PLoS One..

[B44] Cutter A.D. (2013). Integrating phylogenetics, phylogeography and population genetics through genomes and evolutionary theory. Mol. Phylogenet. Evol..

[B45] Dasmahapatra K.K., Lamas G., Simpson F., Mallet J. (2010). The anatomy of a “suture zone” in Amazonian butterflies: a coalescent-based test for vicariant geographic divergence and speciation. Mol. Ecol..

[B46] Day J.J., Peart C.R., Brown K.J., Friel J.P., Bills R., Moritz T. (2013). Continental diversification of an African catfish radiation (Mochokidae: Synodontis). Syst. Biol..

[B47] Degnan J.H., Rosenberg N.A. (2006). Discordance of species trees with their most likely gene trees. PLoS Genet..

[B48] Derryberry E.P., Claramunt S., Derryberry G., Chesser R.T., Cracraft J., Aleixo A., Pérez-Emán J., Remsen J.V., Brumfield R.T. (2011). Lineage diversification and morphological evolution in a large-scale continental radiation: the neotropical ovenbirds and woodcreepers (Aves: Furnariidae). Evolution.

[B49] Drummond A.J., Suchard M.A., Xie D., Rambaut A. (2012). Bayesian phylogenetics with BEAUti and the BEAST 1.7. Mol. Biol. Evol..

[B50] Duenez-Guzman E.A., Mavárez J., Vose M.D., Gavrilets S. (2009). Case studies and mathematical models of ecological speciation. 4. Hybrid speciation in butterflies in a jungle. Evolution.

[B51] Eaton D.A.R., Ree R.H. (2013). Inferring phylogeny and introgression using RADseq data: an example from flowering plants (*Pedicularis*: Orobanchaceae). Syst. Biol..

[B52] Edgar R.C. (2004). MUSCLE: multiple sequence alignment with high accuracy and high throughput. Nucleic Acids Res..

[B53] Edwards S.V. (2009). Is a new and general theory of molecular systematics emerging?. Evolution.

[B54] Edwards S.V., Liu L., Pearl D.K. (2007). High-resolution species trees without concatenation. Proc. Natl. Acad. Sci. USA..

[B55] Elias M., Hill R.I., Willmott K.R., Dasmahapatra K.K., Brower A.V., Mallet J., Jiggins C.D. (2007). Limited performance of DNA barcoding in a diverse community of tropical butterflies. Proc. Biol. Sci..

[B56] Elias M., Gompert Z., Jiggins C., Willmott K. (2008). Mutualistic interactions drive ecological niche convergence in a diverse butterfly community. PLoS Biol..

[B57] Emsley M.G. (1965). Speciation in *Heliconius* (Lep., Nymphalidae): morphology and geographic distribution. Zoologica.

[B58] Etienne R.S., Haegemann B. (2012). A conceptual and statistical framework for adaptive radiations with a key role for diversity dependence. Am. Nat..

[B59] Etienne R.S., Haegeman B., Stadler T., Aze T., Pearson P.N., Purvis A., Phillimore A.B. (2012). Diversity-dependence brings molecular phylogenies closer to agreement with the fossil record. Proc. Biol. Sci..

[B60] Flanagan N.S., Tobler A., Davison A., Pybus O.G., Kapan D.D., Planas S., Linares M., Heckel D., McMillan W.O. (2004). Historical demography of Mullerian mimicry in the neotropical *Heliconius* butterflies. Proc. Natl. Acad. Sci. USA..

[B61] Fulton T.L., Strobeck C. (2009). Multiple markers and multiple individuals refine true seal phylogeny and bring molecules and morphology back in line. Proc. R. Soc. B Biol. Sci..

[B62] Gatesy J., Baker R.H. (2005). Hidden likelihood support in genomic data: can forty-five wrongs make a right?. Syst. Biol..

[B63] Gatesy J., Springer M.S. (2013). Concatenation *versus* coalescence *versus* “concatalescence”. Proc. Natl. Acad. Sci. USA..

[B64] Gerard D., Gibbs H.L., Kubatko L. (2011). Estimating hybridization in the presence of coalescence using phylogenetic intraspecific sampling. BMC Evol. Biol..

[B65] Gilbert L.E., Price P., Lewinsohn T., Fernandes T., Benson W. (1991). Biodiversity of a Central American Heliconius community: pattern, process, and problems. Plant–animal interactions: evolutionary ecology in tropical and temperate regions.

[B66] Glor R.E. (2010). Phylogenetic insights on adaptive radiation. Annu. Rev. Ecol. Evol. Syst..

[B67] Gregory-Wodzicki K.M. (2000). Uplift history of the Central and Northern Andes: a review. Geol. Soc. Am. Bull..

[B68] Hall J.P.W., Harvey D.J. (2002). The phylogeogrpahy of Amazonia revisited: new evidence from Riodinid butterflies. Evolution.

[B69] Heled J., Drummond A.J. (2010). Bayesian inference of species trees from multilocus data. Mol. Biol. Evol..

[B70] Heliconius Genome Consortium (2012). Islands of divergence underlie adaptive radiation in a butterfly genome. Nature.

[B71] Hillis D.M., Heath T.A., St. John K. (2005). Analysis and visualization of tree space. Syst. Biol..

[B72] Hines H.M., Counterman B.A., Papa R., Albuquerque de Moura P., Cardoso M.Z., Linares M., Mallet J., Reed R.D., Jiggins C.D., Kronforst M.R., McMillan W.O. (2011). Wing patterning gene redefines the mimetic history of *Heliconius* butterflies. Proc. Natl. Acad. Sci. USA..

[B73] Holland B.R., Huber K.T., Dress A., Moulton V. (2002). Delta plots: a tool for analyzing phylogenetic distance data. Mol. Biol. Evol..

[B74] Hoorn C., Wesselingh F.P., ter Steege H., Bermudez M.A., Mora A., Sevink J., Sanmartín I., Sanchez-Meseguer A., Anderson C.L., Figueiredo J.P., Jaramillo C., Riff D., Negri F.R., Hooghiemstra H., Lundberg J., Stadler T., Särkinen T., Antonelli A. (2010). Amazonia through time: Andean uplift, climate change, landscape evolution, and biodiversity. Science.

[B75] Jaramillo C., Hoorn C., Silva S.A.F., Leite F., Herrera F., Quiroz L., Rodolfo D., Hoorn C., Wesselingh F.P. (2010). The origin of the modern Amazon rainforest: implications of the palynological and palaeobotanical record. Amazonia, landscape and species evolution: a look into the past.

[B76] Jiggins C.D., Naisbit R.E., Coe R.L., Mallet J. (2001). Reproductive isolation caused by colour pattern mimicry. Nature.

[B77] Jiggins C.D., Mcmillan W.O., King P., Mallet J. (1997). The maintenance of species differences across a *Heliconius* hybrid zone. Heredity.

[B78] Jiggins C.D. (2008). Ecological speciation in mimetic butterflies. BioScience.

[B79] Jiggins C.D., Salazar C., Linares M., Mavarez J. (2008). Review. Hybrid trait speciation and *Heliconius* butterflies. Philos. Trans. R. Soc. Lond. B Biol. Sci..

[B80] Jones R.T., Le Poul Y., Whibley A.C., Mérot C., Ffrench-Constant R.H., Joron M. (2013). Wing shape variation associated with mimicry in butterflies. Evolution.

[B81] Katoh K. (2002). MAFFT: a novel method for rapid multiple sequence alignment based on fast Fourier transform. Nucleic Acids Res..

[B82] Kloepper T.H., Huson D.H. (2008). Drawing explicit phylogenetic networks and their integration into SplitsTree. BMC Evol. Biol..

[B83] Knowles L., Kubatko L. (2010). Estimating species trees. Estimating species trees: practical and theoretical aspects.

[B84] Kronforst M.R., Hansen M.E.B., Crawford N.G., Gallant J.R., Zhang W., Kulathinal R.J., Kapan D.D., Mullen S.P. (2013). Hybridization reveals the evolving genomic architecture of speciation. Cell Rep..

[B85] Kubatko L., Meng C. (2010). Accommodating hybridization in a multilocus phylogenetic framework. Estimating species trees: practical and theoretical aspects.

[B86] Kunte K., Shea C., Aardema M.L., Scriber J.M., Juenger T.E., Gilbert L.E., Kronforst M.R. (2011). Sex chromosome mosaicism and hybrid speciation among tiger swallowtail butterflies. PLoS Genet..

[B87] Lamas G., Callaghan C., Casagrande M., Mielke O., Pyrcz T., Robbins R., Viloria. A., Heppner J. (2004). Hesperioidea—Papilionoidea. Atlas of neotropical lepidoptera. checklist: Part 4A.

[B88] Lanfear R., Calcott B., Ho S.Y.W., Guindon S. (2012). Partitionfinder: combined selection of partitioning schemes and substitution models for phylogenetic analyses. Mol. Biol Evol.

[B89] Larget B.R., Kotha S.K., Dewey C.N., Ané C. (2010). BUCKy: gene tree/species tree reconciliation with Bayesian concordance analysis. Bioinformatics.

[B90] Langham G.M. (2004). Specialized avian predators repeatedly attack novel color morphs of Heliconius butterflies. Evolution.

[B91] Leaché A.D., Harris R.B., Rannala B., Yang Z. (2014). The influence of gene flow on species tree estimation: a simulation study. Syst. Biol..

[B92] Leaché A.D., Rannala B. (2010). The accuracy of species tree estimation under simulation: a comparison of methods. Syst. Biol..

[B93] Lee C.S., McCool B.A., Moore J.L., Hillis D.M., Gilbert L.E. (1992). Phylogenetic study of Heliconiine butterflies based on morphology and restriction analysis of ribosomal RNA genes. Zool. J. Linn. Soc..

[B94] Lee J.Y., Joseph L., Edwards S.V. (2012). A species tree for the Australo-Papuan Fairy-wrens and allies (Aves: Maluridae). Syst. Biol..

[B95] Leigh J.W., Susko E., Baumgartner M., Roger A.J. (2008). Testing congruence in phylogenomic analysis. Syst. Biol..

[B96] Lewis A.R., Marchant D.R., Ashworth A.C., Hemming S.R., Machlus M.L. (2007). Major middle Miocene global climate change: evidence from East Antarctica and the Transantarctic Mountains. Geol. Soc. Am. Bull..

[B97] Linnen C.R., Farrell B.D. (2008). Comparison of methods for species-tree inference in the sawfly genus *Neodiprion* (Hymenoptera: Diprionidae). Syst. Biol..

[B98] Maddison W.P., Knowles L.L. (2006). Inferring phylogeny despite incomplete lineage sorting. Syst. Biol..

[B99] http://www.mesquiteproject.org.

[B100] Mallet J., Barton N.H. (1989). Strong natural selection in a warning-color hybrid zone. Evolution.

[B101] Mallet J., Beltrán M., Neukirchen W., Linares M. (2007). Natural hybridization in heliconiine butterflies: the species boundary as a continuum. BMC Evol. Biol..

[B102] Mallet J., Gilbert L.E. (1995). Why are there so many mimicry rings? Correlations between habitat, behaviour and mimicry in Heliconius butterflies. Biol. J. Linn. Soc..

[B103] Mallet J., Joron M. (1999). Evolution of diversity in warning color and mimicry: polymorphisms, shifting balance, and speciation. Annu. Rev Ecol Syst.

[B104] Mallet J., McMillan W., Jiggins C., Howard D., Berlocher S. (1998). Mimicry and warning color at the boundary between races and species. Endless forms: species and speciation.

[B105] Martin A., Papa R., Nadeau N.J., Hill R.I., Counterman B.A., Halder G., Jiggins C.D., Kronforst M.R., Long A.D., McMillan W.O., Reed R.D. (2012). Diversification of complex butterfly wing patterns by repeated regulatory evolution of a Wnt ligand. Proc. Natl. Acad. Sci. USA..

[B106] Martin S.H., Dasmahapatra K.K., Nadeau N.J., Salazar C., Walters J.R., Simpson F., Blaxter M., Manica A., Mallet J., Jiggins C.D. (2013). Genome-wide evidence for speciation with gene flow in *Heliconius* butterflies. Genome Res..

[B107] Massardo D., Fornel R., Kronforst M., Gonçalves G.L., Moreira G.R.P. (2014). Diversification of the silverspot butterflies (Nymphalidae) in the Neotropics inferred from multi-locus DNA sequences. Mol. Phylogenet. Evol..

[B108] Mavárez J., Salazar C.A., Bermingham E., Salcedo C., Jiggins C.D., Linares M. (2006). Speciation by hybridization in *Heliconius* butterflies. Nature.

[B109] Mérot C., Mavárez J., Evin A., Dasmahapatra K.K., Mallet J., Lamas G., Joron M. (2013). Genetic differentiation without mimicry shift in a pair of hybridizing *Heliconius* species (Lepidoptera: Nymphalidae). Biol. J. Linn. Soc..

[B110] Merrill R.M., Gompert Z., Dembeck L.M., Kronforst M.R., McMillan W.O., Jiggins C.D. (2011). Mate preference across the speciation continuum in a clade of mimetic butterflies. Evolution.

[B111] Merrill R.M., Naisbit R.E., Mallet J., Jiggins C.D. (2013). Ecological and genetic factors influencing the transition between host-use strategies in sympatric *Heliconius* butterflies. J. Evol. Biol..

[B112] Merrill R.M., Wallbank R.W.R., Bull V., Salazar P.C.A., Mallet J., Stevens M., Jiggins C.D. (2012). Disruptive ecological selection on a mating cue. Proc. Biol. Sci..

[B113] Moen D., Morlon H. (2014). From dinosaurs to modern bird diversity: extending the time scale of adaptive radiation. PLoS Biol.

[B114] Moreira G.R.P., Mielke C.G.C. (2010). A new species of *Neruda* Turner, 1976 from northeast Brazil (Lepidoptera: Nymphalidae, Heiconiinae, Heliconiini). Nachrichten des Entomol. Vereins Apollo..

[B115] Mullen S.P., Savage W.K., Wahlberg N., Willmott K.R. (2011). Rapid diversification and not clade age explains high diversity in neotropical Adelpha butterflies. Proc. Biol. Sci. R Soc.

[B116] Müller C.J., Beheregaray L.B. (2010). Palaeo island-affinities revisited—biogeography and systematics of the Indo-Pacific genus *Cethosia* Fabricius (Lepidoptera: Nymphalidae). Mol. Phylogenet. Evol..

[B117] Nadeau N.J., Martin S.H., Kozak K.M., Salazar C., Dasmahapatra K.K., Davey J.W., Baxter S.W., Blaxter M.L., Mallet J., Jiggins C.D. (2013). Genome-wide patterns of divergence and gene flow across a butterfly radiation. Mol. Ecol..

[B118] http://www.abc.se/~nylander/.

[B119] Paradis E., Claude J., Strimmer K. (2004). APE: analyses of phylogenetics and evolution in R language. Bioinformatics.

[B120] Pardo-Diaz C., Salazar C., Baxter S.W., Mérot C., Figueiredo-Ready W., Joron M., McMillan W.O., Jiggins C.D. (2012). Adaptive introgression across species boundaries in *Heliconius* butterflies. PLoS Genet..

[B121] Penney H.D., Hassall C., Skevington J.H., Abbott K.R., Sherratt T.N. (2012). A comparative analysis of the evolution of imperfect mimicry. Nature..

[B122] Penz C. (1999). Higher level phylogeny for the passion-vine butterflies (Nymphalidae, Heliconiinae) based on early stage and adult morphology. Zool. J. Linn. Soc..

[B123] Penz C.M., Peggie D. (2003). Phylogenetic relationships among Heliconiinae genera based on morphology (Lepidoptera: Nymphalidae). Syst. Entomol..

[B124] Pfennig D. (2012). Mimicry: ecology, evolution, and development. Curr. Zool..

[B125] Plummer M., Best N., Cowles K., Vines K. (2006). CODA: convergence diagnosis and output analysis for MCMC. R News.

[B126] Pohl N., Sison-Mangus M.P., Yee E.N., Liswi S.W., Briscoe A.D. (2009). Impact of duplicate gene copies on phylogenetic analysis and divergence time estimates in butterflies. BMC Evol. Biol..

[B127] Posada D., Crandall K.A. (1998). Bioinformatics applications note. MODELTEST: testing the model of DNA substitution. Evolution.

[B128] Pybus O.G., Harvey P.H. (2000). Testing macro-evolutionary models using incomplete molecular phylogenies. Proc. Biol. Sci..

[B129] Quek S.-P., Counterman B.A., Albuquerque de Moura P., Cardoso M.Z., Marshall C.R., McMillan W.O., Kronforst M.R. (2010). Dissecting comimetic radiations in *Heliconius* reveals divergent histories of convergent butterflies. Proc. Natl. Acad. Sci. USA..

[B130] http://www.r-project.org.

[B131] Rabosky D.L. (2014). Automatic detection of key innovations, rate shifts, and diversity-dependence on phylogenetic trees. PLoS One.

[B132] Rabosky D.L., Donnellan S.C., Grundler M., Lovette I.J. (2014a). Analysis and visualization of complex macroevolutionary dynamics: an example from Australian scincid lizards. Syst. Biol..

[B133] Rabosky D.L., Grundler M., Anderson C., Title P., Shi J.J., Brown J.W., Larson J.G. (2014b). BAMMtools: an R package for the analysis of evolutionary dynamics on phylogenetic trees. Methods Ecol. Evol..

[B134] http://www.tree.bio.ed.ac.uk/software/figtree/.

[B135] http://beast.bio.ed.ac.uk/.

[B136] Reid N.M., Hird S.M., Brown J.M., Pelletier T.A., McVay J.D., Satler J.D., Carstens B.C. (2013). Poor fit to the multispecies coalescent is widely detectable in empirical data. Syst. Biol..

[B137] Robinson D.F., Foulds L.R. (1981). Comparison of phylogenetic trees. Math. Biosci..

[B138] Ronquist F., Huelsenbeck J.P. (2003). MrBayes 3: Bayesian phylogenetic inference under mixed models. Bioinformatics.

[B139] Rosser N., Phillimore A.B., Huertas B., Willmott K.R., Mallet J. (2012). Testing historical explanations for gradients in species richness in Heliconiine butterflies of tropical America. Biol. J. Linn. Soc..

[B140] Roure B., Baurain D., Philippe H. (2013). Impact of missing data on phylogenies inferred from empirical phylogenomic data sets. Mol. Biol. Evol..

[B141] Rull V. (2011). Neotropical biodiversity: timing and potential drivers. Trends Ecol. Evol..

[B142] Salazar C., Baxter S.W., Pardo-Diaz C., Wu G., Surridge A., Linares M., Bermingham E., Jiggins C.D. (2010). Genetic evidence for hybrid trait speciation in *Heliconius* butterflies. PLoS Genet..

[B143] Salzberg S.L., Phillippy A.M., Zimin A., Puiu D., Magoc T., Koren S., Treangen T.J., Schatz M.C., Delcher A.L., Roberts M., Marçais G., Pop M., Yorke J.A. (2012). GAGE: a critical evaluation of genome assemblies and assembly algorithms. Genome Res..

[B144] Sauquet H., Ho S.Y.W., Gandolfo M.A., Jordan G.J., Wilf P., Cantrill D.J., Bayly M.J., Bromham L., Brown G.K., Carpenter R.J., Lee D.M., Murphy D.J., Sniderman J.M., Udovicic F. (2012). Testing the impact of calibration on molecular divergence times using a fossil-rich group: the case of Nothofagus (Fagales). Syst. Biol..

[B145] Savage W.K., Mullen S.P. (2009). A single origin of Batesian mimicry among hybridizing populations of admiral butterflies (*Limenitis arthemis*) rejects an evolutionary reversion to the ancestral phenotype. Proc. Biol. Sci..

[B146] Schluter D. (2000). The ecology of adaptive radiation.

[B147] Schwarz G. (1978). Estimating the dimension of a model. Ann. Stat..

[B148] Sheppard P.M., Turner J.R.G., Brown K.S., Benson W.W., Singer M.C. (1985). Genetics and the evolution of Müllerian mimicry in *Heliconius* butterflies. Philos. Trans. R. Soc. B Biol. Sci..

[B149] Sherratt T.N. (2008). The evolution of Müllerian mimicry. Naturwissenschaften.

[B150] Shimodaira H., Hasegawa M. (1989). Multiple comparisons of log-likelihoods with applications to phylogenetic inference. DNA Seq..

[B151] Simpson J.T., Wong K., Jackman S.D., Schein J.E., Jones S.J.M., Birol I. (2009). ABySS: a parallel assembler for short read sequence data. Genome Res..

[B152] Smith B.T., Harvey M.G., Faircloth B.C., Glenn T.C., Brumfield R.T. (2013). Target capture and massively parallel sequencing of Ultraconserved Elements for comparative studies at shallow evolutionary timescales. Syst. Biol..

[B153] Solomon S.E., Bacci M., Martins J., Vinha G.G., Mueller U.G. (2008). Paleodistributions and comparative molecular phylogeography of leafcutter ants (*Atta* spp.) provide new insight into the origins of Amazonian diversity. PLoS One.

[B154] Song S., Liu L., Edwards S.V., Wu S. (2012). Resolving conflict in Eutherian mammal phylogeny using phylogenomics and the multispecies coalescent model. Proc. Natl. Acad. Sci. USA..

[B155] Stadler T. (2013). Recovering speciation and extinction dynamics based on phylogenies. J. Evol. Biol..

[B156] Stamatakis A. (2006). RAxML-VI-HPC: maximum likelihood-based phylogenetic analyses with thousands of taxa and mixed models. Bioinformatics.

[B157] Stamatakis A. (2014). RAxML version 8: a tool for phylogenetic analysis and post-analysis of large phylogenies. Bioinformatics (Oxford, England).

[B158] Steel M., Velasco J.D. (2014). Axiomatic opportunities and obstacles for inferring a species tree from gene trees. Syst. Biol..

[B159] Strimmer K., Rambaut A. (2002). Inferring confidence sets of possibly misspecified gene trees. Proc. Biol. Sci..

[B160] Supple M.A., Hines H.M., Dasmahapatra K.K., Lewis J.J., Nielsen D.M., Lavoie C., Ray D.A., Salazar C., McMillan W.O., Counterman B.A. (2013). Genomic architecture of adaptive color pattern divergence and convergence in *Heliconius* butterflies. Genome Res..

[B161] http://www.paup.csit.fsu.edu.

[B162] Than C., Ruths D., Nakhleh L. (2008). PhyloNet: a software package for analyzing and reconstructing reticulate evolutionary relationships. BMC Bioinformatics.

[B163] Turchetto-Zolet A.C., Pinheiro F., Salgueiro F., Palma-Silva C. (2013). Phylogeographical patterns shed light on evolutionary process in South America. Mol. Ecol..

[B164] Turner J. (1965). Evolution of complex polymorphism and mimicry in distasteful South American butterflies. Proc. XII Int. Cong. Entomol. London..

[B165] Turner J.R., Johnson M.S., Eanes W.F. (1979). Contrasted modes of evolution in the same genome: allozymes and adaptive change in Heliconius. Proc. Natl. Acad. Sci. USA..

[B166] Vamosi S.M. (2005). On the role of enemies in divergence and diversification of prey: a review and synthesis. Can. J. Zool..

[B167] van Velzen R., Wahlberg N., Sosef M.S.M., Bakker F.T. (2013). Effects of changing climate on species diversification in tropical forest butterflies of the genus Cymothoe (Lepidoptera: Nymphalidae). Biol. J. Linn. Soc..

[B168] Wahlberg N., Leneveu J., Kodandaramaiah U., Peña C., Nylin S., Freitas A.V.L., Brower A.V.Z. (2009). Nymphalid butterflies diversify following near demise at the Cretaceous/Tertiary boundary. Proc. Biol. Sci..

[B169] Wahlberg N., Wheat C.W. (2008). Genomic outposts serve the phylogenomic pioneers: designing novel nuclear markers for genomic DNA extractions of lepidoptera. Syst. Biol..

[B170] Wiens J.J., Morrill M.C. (2011). Missing data in phylogenetic analysis: reconciling results from simulations and empirical data. Syst. Biol..

[B171] Wright J.J. (2011). Conservative coevolution of Müllerian mimicry in a group of rift lake catfish. Evolution.

[B172] Xia X., Xie Z. (2001). DAMBE: software package for data analysis in molecular biology and evolution. J. Hered..

[B173] Yu Y., Than C., Degnan J.H., Nakhleh L. (2011). Coalescent histories on phylogenetic networks and detection of hybridization despite incomplete lineage sorting. Syst. Biol..

[B174] Zhang J., Kapli P., Pavlidis P., Stamatakis A. (2013a). A general species delimitation method with applications to phylogenetic placements. Bioinformatics.

[B175] Zhang W., Kunte K., Kronforst M.R. (2013b). Genome-wide characterization of adaptation and speciation in tiger swallowtail butterflies using *de novo* transcriptome assemblies. Genome Biol. Evol..

